# Mutations of N1 Riboswitch Affect its Dynamics and Recognition by Neomycin Through Conformational Selection

**DOI:** 10.3389/fmolb.2021.633130

**Published:** 2021-02-18

**Authors:** Piotr Chyży, Marta Kulik, Suyong Re, Yuji Sugita, Joanna Trylska

**Affiliations:** ^1^ Centre of New Technologies, University of Warsaw, Warsaw, Poland; ^2^ Faculty of Physics, University of Warsaw, Warsaw, Poland; ^3^ Department of Chemistry, Biological and Chemical Research Centre, University of Warsaw, Warsaw, Poland; ^4^ RIKEN Center for Biosystems Dynamics Research (BDR), Kobe, Japan; ^5^ National Institutes of Biomedical Innovation, Health and Nutrition, Ibaraki, Japan; ^6^ RIKEN Cluster for Pioneering Research (CPR), Wako, Japan; ^7^ RIKEN Center for Computational Science, Kobe, Japan

**Keywords:** RNA, riboswitch, aminoglycosides, neomycin, molecular dynamics simulations, replica exchange with solute tempering

## Abstract

Short, structured fragments of non-coding mRNA may act as molecular switches upon binding specific ligands, regulating the translation of proteins encoded downstream this mRNA sequence. One switch, called riboswitch N1, is regulated by aminoglycosides such as neomycin. Nucleobase mutations in the apical loop, although distant from the binding pocket, significantly affect neomycin affinity and riboswitch regulatory efficiency. To explain this influence, we conducted molecular dynamics simulations using generalized replica exchange with solute tempering (gREST). Translation assay of a reporter protein in a yeast system shows that mutating A17 to G in the riboswitch apical loop reduces 6-fold the translation regulation efficiency of the mutant. Indeed, simulations of the unbound riboswitch show that G17 frequently stacks with base 7, while base 8 is stabilized towards the binding site in a way that it may interfere with the conformational selection mechanism and decrease riboswitch regulatory activity. In the riboswitch complexes, this single-point A to G mutation disrupts a strong hydrogen bond between nucleotides 5 and 17 and, instead, a new hydrogen bond between residue 17 and neomycin is created. This change forces neomycin to occupy a slightly shifted position in the binding pocket, which increases neomycin flexibility. Our simulations of the U14C mutation suggest that the riboswitch complex with neomycin is more stable if cytosine 14 is protonated. A hydrogen bond between the RNA phosphate and protonated cytosine appears as the stabilizing factor. Also, based on the cell-free translation assay and isothermal titration calorimetry experiments, mutations of nucleotides 14 and 15 affect only slightly the riboswitch ability to bind the ligand and its activity. Indeed, the simulation of the unbound U15A mutant suggests conformations preformed for ligand binding, which may explain slightly higher regulatory activity of this mutant. Overall, our results corroborate the *in vivo* and *in vitro* experiments on the N1 riboswitch-neomycin system, detail the relationship between nucleobase mutations and RNA dynamics, and reveal the conformations playing the major role in the conformational selection mechanism.

## 1 Introduction

Riboswitches are regulatory RNA elements, typically found in 5ʹ-untranslated regions (5ʹ-UTR) of mRNA and positioned upstream of the regulated coding sequence. They control gene expression by directly binding ions and metabolites (McCown et al., 2017) or responding to changes in pH or temperature (Bastet et al., 2011). Since additional protein factors are not necessary for their activity, riboswitches are a valuable tool for gene regulation in synthetic biology (Serganov and Nudler, 2013; Berens et al., 2015; Sinumvayo et al., 2018). For example, two riboswitches can be engineered to form a NOR logic gate (Boussebayle et al., 2019).

Riboswitches that bind ligands typically contain a ligand binding sensory domain (aptamer) and expression platform. Interactions between the aptamer and ligand determine the conformation of the expression platform. Structural rearrangements of this platform upon ligand binding influence the expression of genes located downstream the same mRNA (Berens et al., 2015). Regulation of gene expression or repression may occur at different levels: transcription, translation, splicing or mRNA cleavage.

Aptamers are single stranded oligonucleotides that selectively bind small ligands. They have been designed even before the discovery of the first natural riboswitch. Aptamers binding a specific ligand are usually isolated by an *in vitro* evolution and selection process (SELEX) (Ellington and Szostak, 1990) involving screening large libraries of nucleic acid oligomers (Banerjee and Nilsen-Hamilton, 2013). Since aptamers are the regulatory parts of riboswitches, they could be designed to constitute synthetic riboswitches. The activities of such synthetic riboswitches can be tested, e.g., by introducing the aptamer to the 5ʹ-UTR of mRNA in a cell or cell-free system producing a reporter protein. Then, the regulatory mechanism of the riboswitch is due to mechanical blocking of scanning the mRNA by the small ribosomal subunit (Etzel and Mörl, 2017).

**FIGURE 1:**
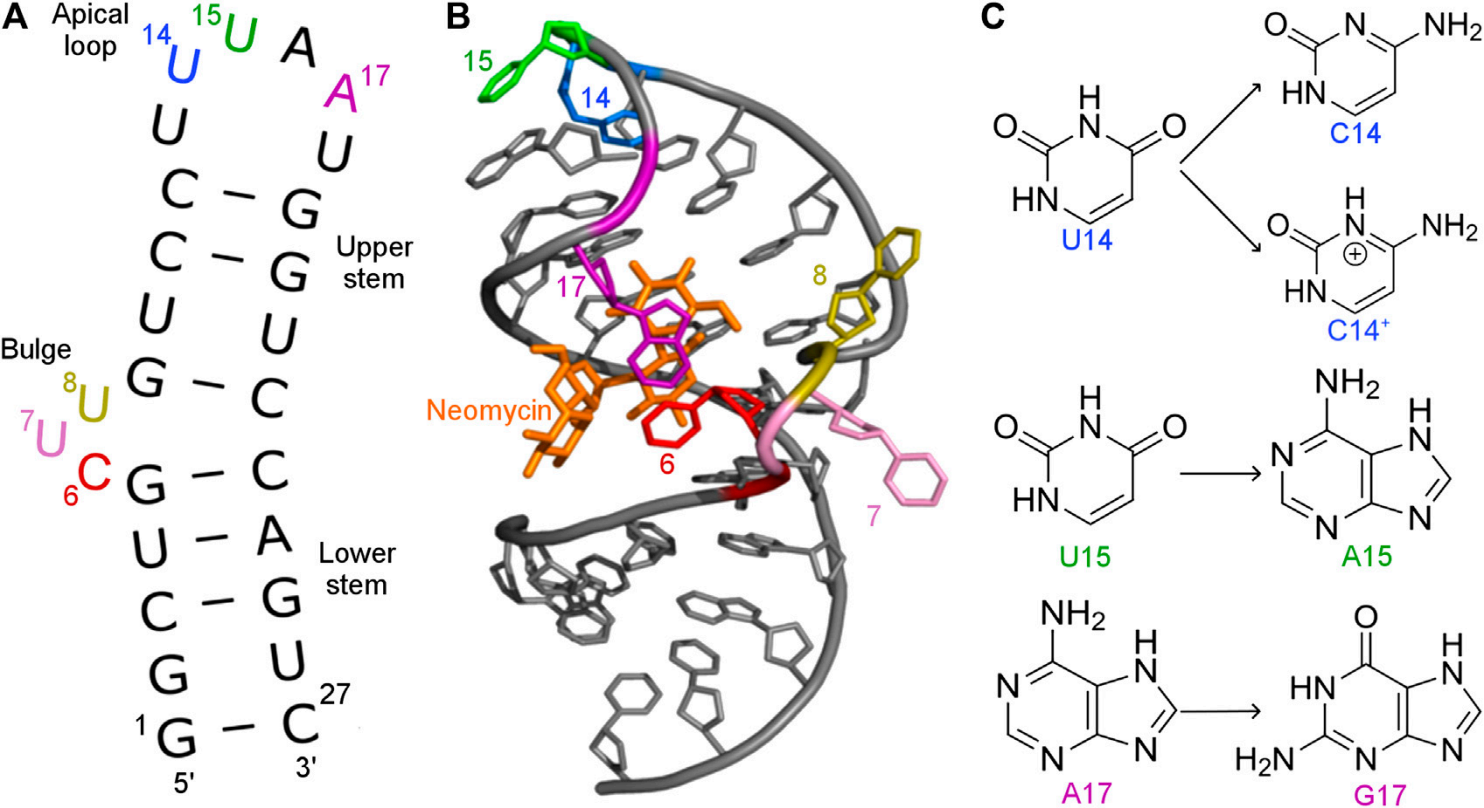
**(A)** Secondary and **(B)** tertiary structure of the N1 riboswitch in the complex with ribostamycin (PDB ID: 2KXM (Duchardt-Ferner et al., 2010)). **(C)** Mutations of nucleobases 14, 15, and 17 of the N1 riboswitch.

A synthetic aminoglycoside-sensing N1 riboswitch (Weigand et al., 2008) was also discovered through screening of aptamers that bind aminoglycoside antibiotics. The N1 riboswitch is a 27-nucleotide-long RNA and is the smallest synthetic riboswitch found active *in vivo* (Gottstein-Schmidtke et al., 2014). Its activity is induced by binding neomycin and was confirmed both *in vitro* and *in vivo* (Weigand et al., 2008). This riboswitch forms a hairpin with two flexible functional regions: a bulge and apical loop (Figure 1). Apart from neomycin, N1 riboswitch binds also paromomycin, tobramycin and ribostamycin. The N1 structure in the complex with ribostamycin (Duchardt-Ferner et al., 2010) and paromomycin (Duchardt-Ferner et al., 2016) was determined by NMR spectroscopy.

The N1 riboswitch is active in yeast if inserted into the 5ʹ-UTR of an mRNA of a reporter gene (Weigand et al., 2008) expressing green fluorescent protein. Fluorescence measurements showed that the expression of green fluorescent protein in yeast depends on the presence of neomycin that supposedly binds the N1 riboswitch inserted into 5ʹ-UTR. Furthermore, the N1 riboswitch works also for ribostamycin though with slightly lower regulatory activity (Weigand et al., 2008). Surprisingly, paromomycin does not inhibit gene expression although it differs from neomycin by only one chemical group, namely, amino versus hydroxyl at 6ʹ position of ring I (Supplementary Figure S1). Using molecular dynamics simulations with replica exchange (Kulik et al., 2018), we have previously proposed the reasons for different activities of these ligands by comparing the dynamics of their complexes. As shown in Table 1, dissociation constants for neomycin, ribostamycin and paromomycin to the isolated riboswitch determined using isothermal titration calorimetry (ITC) (Duchardt-Ferner et al., 2016; Weigand et al., 2014) agree with the translation blocking efficiencies obtained *in vivo*.

**TABLE 1:** Dissociation constants (K_*d*_) determined from ITC experiments and regulatory factors (R.f.) calculated as the ratio of relative fluorescence of GFP expression in the absence and presence of neomycin (Weigand et al., 2014; Duchardt-Ferner et al., 2016). The smaller the regulatory factor, the lower the activity of the riboswitch.

	Ribostamycin		Neomycin		Paromomycin	
	K_*d*_ [nM]	R.f.	K_*d*_ [nM]	R.f.	K_*d*_ [nM]	R.f
N1	330 ± 30	2.3	9.2 ± 1.3	8.6	5130 ± 26	1.0
U14C	−	−	24.2 ± 1.5	3.0	−	−
U15A	−	−	11.0± 0.3	10.0	−	−
A17G	−	−	130.9 ± 16.8	1.5	−	−
A17C	1530 ± 140	1.1	−	2.9	11890 ± 1550	1.0

Stopped-flow fluorescence assays of the N1 riboswitch labeled with cytidine analog at positions 6 and 8 showed that neomycin binding kinetics is fast and proceeds according to the two-step binding model, in which the initial non-specific ligand binding is followed by a specific binding with minor conformational changes. Also, the study identified the conformational selection mechanism as dominating in the neomycin binding (Gustmann et al., 2019).

Several mutations were introduced in the isolated N1 riboswitch to elucidate the importance of the aptamer sequence for aminoglycoside binding. Mutations of C6, U7, and U8 nucleotides revealed that the sequence of the bulge is crucial for the riboswitch activity (Weigand et al., 2011). Various mutations in the apical loop also hinder riboswitch activity, apart from U15A (Weigand et al., 2014). Mutational analysis of several minimal neomycin aptamers similar to N1 have shown that the formation of the upper stem, between the bulge and apical loop (Figure 1), is critical for regulatory properties of the riboswitch, while the sequence of the lower stem may be gently modified without the loss of function (Weigand et al., 2008). Apart from activity studies, also dissociation constants show that N1 mutations affect ligand binding (Table 1). For example, the dissociation constant of neomycin - U14C mutant is two-fold smaller than of neomycin - N1 riboswitch, although regulatory activity of the U14C mutant in yeast is preserved (Weigand et al., 2014) (the relative expression of green fluorescent protein with and without neomycin is similar as for the non-mutated N1 riboswitch). The experimental K_*d*_ of neomycin for the U15A mutant is close to the reference N1 riboswitch, but the measured regulatory activity of the U15A mutant in yeast is even better. Finally, the A17G change drastically increases K_*d*_ and decreases the riboswitch regulatory activity.

To elucidate the link between the nucleobase mutations, riboswitch dynamics and ligand binding, we performed generalized replica exchange with solute tempering (gREST) simulations of selected riboswitch mutants and their complexes with neomycin. Specifically, we explored the dynamics of the A17G mutant, which affects the riboswitch regulatory activity and K_*d*_ to the largest extent. For the U14C mutant, we investigated why in the neomycin complex the protonated cytosine state is preferred over the deprotonated state (Weigand et al., 2014). Lastly, we determined the conformational ensemble of N1 and the U15A mutant in unbound forms to verify possible enrichment of riboswitch states preformed for neomycin binding, as suggested by experiments (Weigand et al., 2014).

## 2 Materials and Methods

### 2.1 Enhanced sampling methods

Parallel simulations of system copies at different temperatures in replica-exchange molecular dynamics (REMD) (Sugita and Okamoto, 1999) increase the sampling of conformations, which is crucial for elucidating the dynamics of flexible RNA systems. In our previous work, we applied the REMD method with 32 replicas to N1 riboswitch with different ligands and confirmed a significant gain in conformational sampling as compared to classical MD (Kulik et al., 2018). Here, to reduce the number of replicas without losing the sampling efficiency, we employ a recently developed method, called generalized replica exchange with solute tempering (gREST) (Kamiya and Sugita, 2018). This method stems from REST or REST2 (Wang et al., 2011; Liu et al., 2005; Moors et al., 2011; Terakawa et al., 2011), in which the acceptance probability for the replica exchange events does not depend on the number of explicit water molecules in the system. This is achieved by dividing the system into a pre-defined solute region, in which the temperature is exchanged between replicas, and the solvent region, kept at room temperature during the simulations. In gREST, the solute selection strategy is more flexible and the solute can include parts of molecules and selected potential energy function terms, such as the dihedral-angle term.

### 2.2 Structure preparation

The structure of N1 riboswitch in the complex with paromomycin was taken from the RCSB Protein Data Bank (PDB ID: 2MXS (Duchardt-Ferner et al., 2016)). Paromomycin was changed to neomycin by replacing 6ʹ-OH with ammonium group (Supplementary Figure S1). This change does not influence the binding pose of the ligand as a similar binding pose is also adopted by ribostamycin in the NMR structure with PDB ID: 2N0J (Duchardt-Ferner et al., 2016). Paromomycin, ribostamycin and neomycin are similar, except for a different substituent at 6ʹ position of ring I (paromomycin: 6ʹ-OH, ribostamycin: 6ʹ-${\text{NH}}_{3}^{+}$, neomycin: 6ʹ-${\text{NH}}_{3}^{+}$) and lack of ring IV in ribostamycin that is present in paromomycin and neomycin. Since paromomycin and ribostamycin exhibit similar binding mode in NMR-derived N1 structures, then neomycin, the combination of the former two, should have a similar mode. The mutant structures were prepared using Pymol (Schrödinger, LLC, 2010) by replacing appropriate nucleobases. Since the unbound riboswitch structures were not determined experimentally, we built them by deleting the ligands. All amino groups in neomycin were protonated (Kulik et al., 2018). Structure optimization and calculation of ESP charges of neomycin were carried out in Gaussian 09 with HF/6-31G* level of theory (Gaussian Inc., 2009). Initial structures were solvated with a 20 Å shell of TIP3P water molecules around RNA (Jorgensen et al., 1983). 52 Na^+^ and 26 Cl^−^ ions were added using the LEaP program from Amber14 (Case et al., 2014) to neutralize each unbound system and achieve 100 mM NaCl concentration, which is the salt concentration used in the UV-melting experiments of the N1 riboswitch (Weigand et al., 2011). For the systems with neomycin, 46 Na^+^ and 26 Cl^−^ ions were added, while when cytosine was protonated, one more Cl^−^ ion was necessary. The number of added ions was calculated based on the number of water molecules in the system. The ion parameters were taken from (Joung and Cheatham, 2008). Even though the Mg^2+^ ions play an important role in the RNA structure-function (Hayes et al., 2012; Roy et al., 2017a, Roy et al., 2017b, Roy et al., 2019) and were present in the NMR experiments, their positions were not determined so they were not considered in the simulations. GAFF (Wang et al., 2004) parameters for neomycin were used together with Amber ff99 (Cornell et al., 1995; Wang et al., 2000) force field for RNA. The force field parameters for neomycin were validated previously in (Kulik et al., 2018). The parmbsc0 (Pérez et al., 2007) and χOL3 (Zgarbova et al., 2011) corrections were applied to the RNA force field (Šponer et al., 2018), previously used in the REMD simulations of RNA (Kulik et al., 2018; Bergonzo et al., 2014).

### 2.3 MD and gREST simulations

Energy minimizations, MD and gREST simulations were run with SPDYN from the GENESIS (v. 1.3 and 1.4) suite of programs (Jung et al., 2015; Kobayashi et al., 2017). The particle mesh Ewald method (Essmann et al., 1995) was used to calculate long-range electrostatic interactions. Lennard-Jones interactions were truncated at 12 Å cutoff distance and the pair list distance set to 13.5 Å. Water molecules were kept rigid with the SETTLE algorithm (Miyamoto and Kollman, 1992). Energy minimizations were carried out for 5000 steps with steepest descent algorithm with positional restraints of 10 kcal/mol/Å^2^ on heavy atoms of RNA and aminoglycosides. The systems were equilibrated at 310.15 K for 3 ns in the NVT ensemble with positional restraints gradually decreasing every 500 ps, which was followed by additional 2 ns simulation in the NPT ensemble without restraints. The atomic coordinates were saved after the last round of equilibrations to use them as the starting point for the gREST procedure. Next, for each system, a 30 ns MD production simulation in the NPT ensemble was performed to calculate the average box size for gREST simulations. The 100‐ns MD production simulation was done in the NVT ensemble. The previously saved atomic coordinates were parametrized in LEaP (Case et al., 2014), energy minimized with the new box size with 5.0 kcal/mol/Å^2^ harmonic restraints on positions of heavy atoms of RNA and aminoglycosides, with cutoff distance of Lennard-Jones interactions at 10 Å and Verlet pair list distance at 11.5 Å. Subsequently, the restart files were used in gREST equilibration in the NVT ensemble for 150 ps with 5 kcal/mol/Å^2^ positional restraints and in the NPT ensemble for 150 ps without restraints. Next, in the gREST production, lasting 300 ns, 8 replicas were used with exchange attempts every 2000 steps. The solute region was defined as RNA, aminoglycoside, counterions added to neutralize the system, and the dihedrals, Coulombic and Lennard-Jones potential energy terms. The temperatures of the solute region ranged from 310.15 K to 370.00 K, while the rest of the system was calculated at 310.15 K (referred to as 310 K hereafter). The RESPA integrator (Tuckerman et al., 1992) with 2.5 fs time step and SHAKE algorithm (Ryckaert et al., 1977) were applied to effectively describe bonds with hydrogens. In conventional MD simulations Langevin thermostat and barostat (Quigley and Probert, 2004) were used, but, in gREST, Bussi temperature and pressure control were applied (Bussi et al., 2007, Bussi et al., 2009). The production simulations are gathered in Supplementary Table S1. The level of convergence of the gREST simulations was verified by calculating the overlap between the covariant matrices of the final gREST trajectory fragments at 310 K (Supplementary Table S2). The overlap of the potential energy of replicas and the random walk in the temperature space in Supplementary Figure S2 confirm that replicas visit the full range of temperatures. The acceptance ratio is at least 17% (Supplementary Table S3) and is sufficient for our simulations (Kamiya and Sugita, 2018).

### 2.4 Data analysis

For analysis, only the gREST simulation trajectories at 310 K were used, which is the temperature used in the ITC experiments (Weigand et al., 2014). The root mean square deviation (RMSD) was calculated using GENESIS analysis tool (rmsd_analysis) (Kobayashi et al., 2017) using the initial structure as a reference. RMSD for neomycin was calculated for all ligand atoms including hydrogens, after the least squares fitting of the RNA non-hydrogen atoms to the reference starting structure. The root mean square fluctuations (RMSF) around average atom positions, χ torsion angles (defined by O4′–C1′–N9–C4 atoms) and pseudo-dihedral angles were analyzed in Gromacs 2019.4 (Abraham et al., 2019). The pseudo-dihedral angles were defined according to Song *et al.* (Song et al., 2009) and were calculated to quantify the flipping of bases with respect to the RNA backbone. The k-means algorithm (Forgy, 1965) implemented in the GENESIS analysis tools (Kobayashi et al., 2017) was used for clustering with a 98% convergence level to obtain 10 clusters. Hydrogen bonds and stacking interactions between ligands and RNA were calculated with cpptraj (Roe and Cheatham, 2013) and MINT (Górska et al., 2015). Hydrogen bond criteria were: the maximum of 3.5 Å as the distance between non-hydrogen atoms and the minimum of 150° for the acceptor-hydrogen-donor angle. First 50 ns of each gREST simulation was omitted in the analysis, except for the RMSD calculations. If not stated otherwise, only heavy atoms of RNA and aminoglycosides were taken into account. Figures were generated with VMD (Humphrey et al., 1996) and Pymol (Schrödinger, LLC, 2010).

## 3 Results and Discussion

### 3.1 Nucleotide substitutions in the apical loop affect the dynamics of the bulge

**FIGURE 2:**
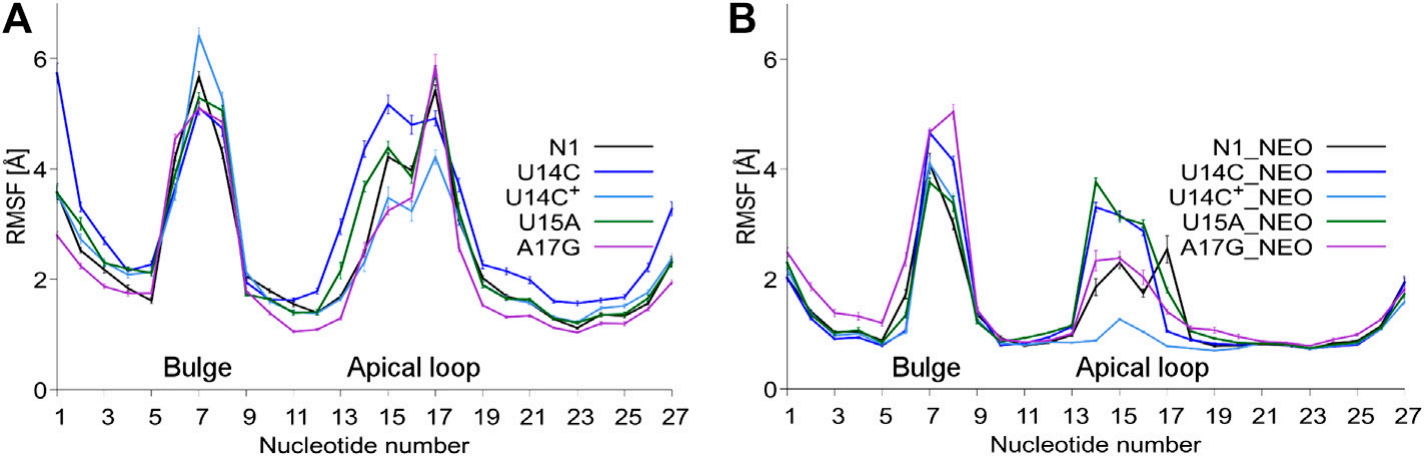
RMSF values per nucleotide at 310 K in: **(A)** unbound and **(B)** neomycin-bound riboswitches. RMSF was calculated for all nucleotide atoms including hydrogen atoms. The block average method was used to calculate the standard error of the mean by dividing the simulation into 10 ns blocks.

The RMSD plots as a function of the simulation time are shown in Supplementary Figure S3. As expected, bound neomycin reduces the RMSD of RNA. In all cases, the RMSD stabilizes after the initial 50 ns of trajectories. Figure 2 shows that consistently the highest RMSF are in the bulge and apical loop regions of riboswitches. The fluctuations of the terminal nucleotides are expected but not considered or discussed because under cellular conditions the termini are connected with other fragments of mRNA. Although mutations were introduced in the apical loop of the riboswitch, the fluctuations in the bulge region also change. For instance, in the bound state, the A17G mutation causes higher fluctuations of the bulge as compared to the unmutated N1 riboswitch. There is also a difference in the apical loop fluctuations between the U14C and U14C^+^ systems both in the unbound and bound states. The amplitude of both RMSD and RMSF values for the N1 riboswitch without mutations corresponds well with the previous results in the temperature REMD simulations (Kulik et al., 2018), implying that similar behaviour of the riboswitch and the same level of conformational sampling have been achieved in both methods.

### 3.2 The C6:A17 contact joins the bulge and apical loop in the riboswitch complexes

**FIGURE 3:**
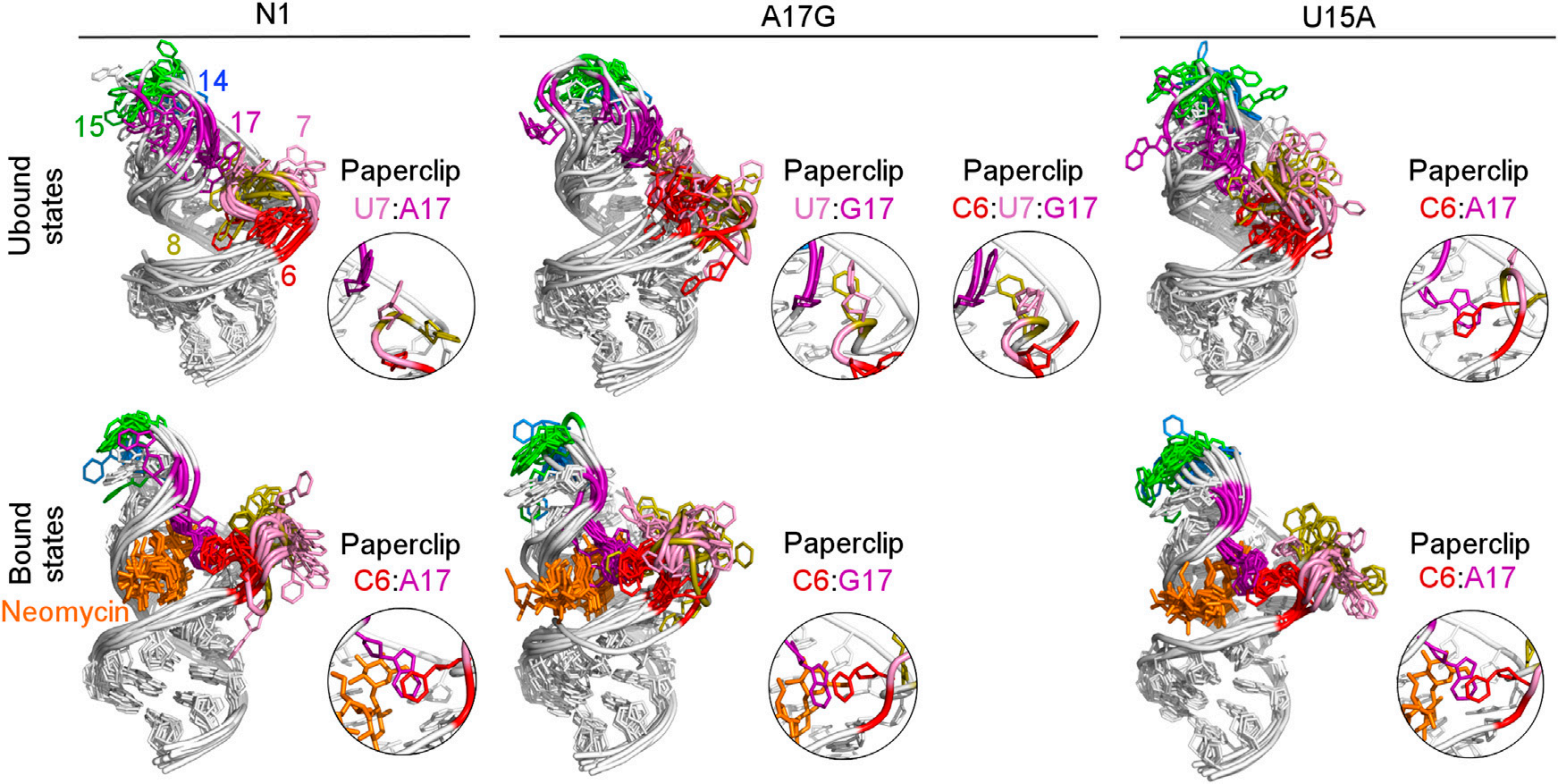
Structures obtained from clustering of N1, A17G and U15A simulations. The paperclips - the van der Waals interactions between the bulge and apical loop seen in at least 25% of simulation frames - are shown in the insets. For other systems, see Supplementary Figure S4.

To examine how the dynamics of the apical loop affects the fluctuations of the bulge we compared the representative structures from gREST simulations (Figure 3 and Supplementary Figure S4). In the unbound systems, all colored bases in the bulge and apical loop acquire various conformations, while the upper and lower stem bases are stably paired. In the simulations with neomycin, U7 and U8 adopt conformations outside the hairpin, while the apical loop nucleotides mostly remain stable due to the C6:A17 stacking. Since this C6:A17 interaction mimics a clip that fastens the bulge and apical loop together, we call it paperclip C6:A17.

The paperclip C6:A17 is rarely found in the trajectories of the unbound riboswitch (Table 2), for which the experimental structures are not known. On the other hand, it is visible in more than 82% of simulation time in all complexes (Table 3). It is also present in all experimental NMR models for the riboswitch complexes with ribostamycin (Duchardt-Ferner et al., 2010) and paromomycin (Duchardt-Ferner et al., 2016). This finding implies that the C6:A17 stacking interaction is mainly formed after neomycin binding and is stabilized by the presence of the ligand. Assuming the two-step binding mechanism (Gustmann et al., 2019), the initial neomycin binding is likely followed by the formation of the C6:A17 paperclip with a minor conformational change that adjusts the binding pocket to the presence of the ligand.

**TABLE 2:** Selected stacking interactions and hydrogen bonds observed in the trajectories of unbound riboswitches with standard deviations (in parentheses) calculated based on the two halves of the trajectory. “–” stands for undetected interaction. The nucleotide sequence is shown in Figure 1. For the full list of interactions, see Supplementary Tables S4–S6.

		Percentage of simulation time [%]				
Base 1	Base 2	N1	U14C	U14C^+^	U15A	A17G
Stacking interactions						
C6	U7	11 (7)	1 (1)	1 (1)	1 (1)	40 (4)
C6	A17	1 (1)	11 (11)	1 (0)	35 (2)	8 (6)
U7	A17	35 (4)	2 (2)	10 (2)	2 (2)	65 (5)
U8	C12	−	1 (1)	1 (1)	7 (1)	54 (4)
Hydrogen bonds						
U8:O2’	C11:N4	−	1 (1)	−	1 (1)	52 (2)
U10:OP2	U8:O2’	−	5 (5)	1 (1)	4 (1)	51 (2)
U13:O4	U8:N3	−	−	−	1 (1)	37 (2)

**TABLE 3:** Selected stacking interactions and hydrogen bonds observed in the trajectories of neomycin-bound riboswitches with standard deviations (in parentheses) calculated based on the two halves of the trajectory. “–” stands for undetected interaction. The nucleotide sequence is shown in Figure 1. For the full list of interactions, see Supplementary Tables S7, S8.

		Percentage of simulation time [%]				
Base 1	Base 2	N1_NEO	U14C_NEO	U14C^+^_NEO	U15A_NEO	A17G_NEO
Stacking interactions						
C6	U7	−	35 (4)	18 (17)	10 (2)	26 (2)
C6	U8	27 (3)	4 (2)	5 (1)	10 (7)	7 (2)
C6	A17	87 (3)	93 (1)	94 (4)	94 (4)	82 (7)
U7	A17	−	9 (1)	5 (4)	2 (1)	25 (14)
Hydrogen bonds						
G5:O2’	A17:N6^*^	70 (1)	76 (2)	85 (0)	57 (3)	1 (0)
G5:OP1	C6:N4	23 (1)	26 (6)	18 (2)	17 (5)	3 (1)
G5:OP1	A17:C2^*^	−	−	−	−	31 (4)
G5:OP2	C6:N4	32 (2)	34 (7)	38 (9)	37 (1)	8 (2)
C6:O2’	U7:OP1	32 (1)	19 (6)	30 (1)	21 (5)	4 (1)
U14:N3	A17:OP2	70 (1)	−	67 (1)	47 (5)	62 (12)

^*^ in the A17G–mutant A17:C2 changes to G17:NH_2_, and A17:N6 to G17:O6.

The most frequent conformations observed in the trajectories of unbound riboswitches (named Cluster 1, Cluster 2, Cluster 3 in Supplementary Figure S5–S9) are characterized by higher structural variety than those in the bound state. The RNA binding pocket is often wide open, without any contacts between the bulge and apical loop. The characteristic paperclip C6:A17 appears in 35% of the simulation frames of the U15A riboswitch, including 17% of the conformations in the second most populated cluster (Figure 3 and Cluster 2 in Supplementary Figure S8A). This paperclip is not found in the representative unbound structures of the N1 riboswitch and U14C, U14C^+^ and A17G mutants.

### 3.3 The A17G mutant in the unbound state forms paperclips involving the U7 base

In the A17G unbound mutant in Clusters 1 and 3 (representing 17 and 16% of the simulation frames, respectively) a strong contact between the bulge U7 and apical loop G17 bases was found, called paperclip U7:G17 (Figure 3 and Supplementary Figure S9A). This paperclip exists in the N1 riboswitch without mutations but it is not common–the U7:A17 stacking is seen in 35% of simulation frames (Table 2). On the other hand, the U7:G17 stacking interaction is present in 65% of frames. Additional van der Waals interaction with C6, visible in Cluster 3 of the A17G mutant, forms a triple stacking interaction, named paperclip C6:U7:G17. This interaction is present for 25% of simulation time in the A17G system and was not detected in other systems (Supplementary Table S5). These paperclips break and form in time, as illustrated in Supplementary Figure S10.

**FIGURE 4:**
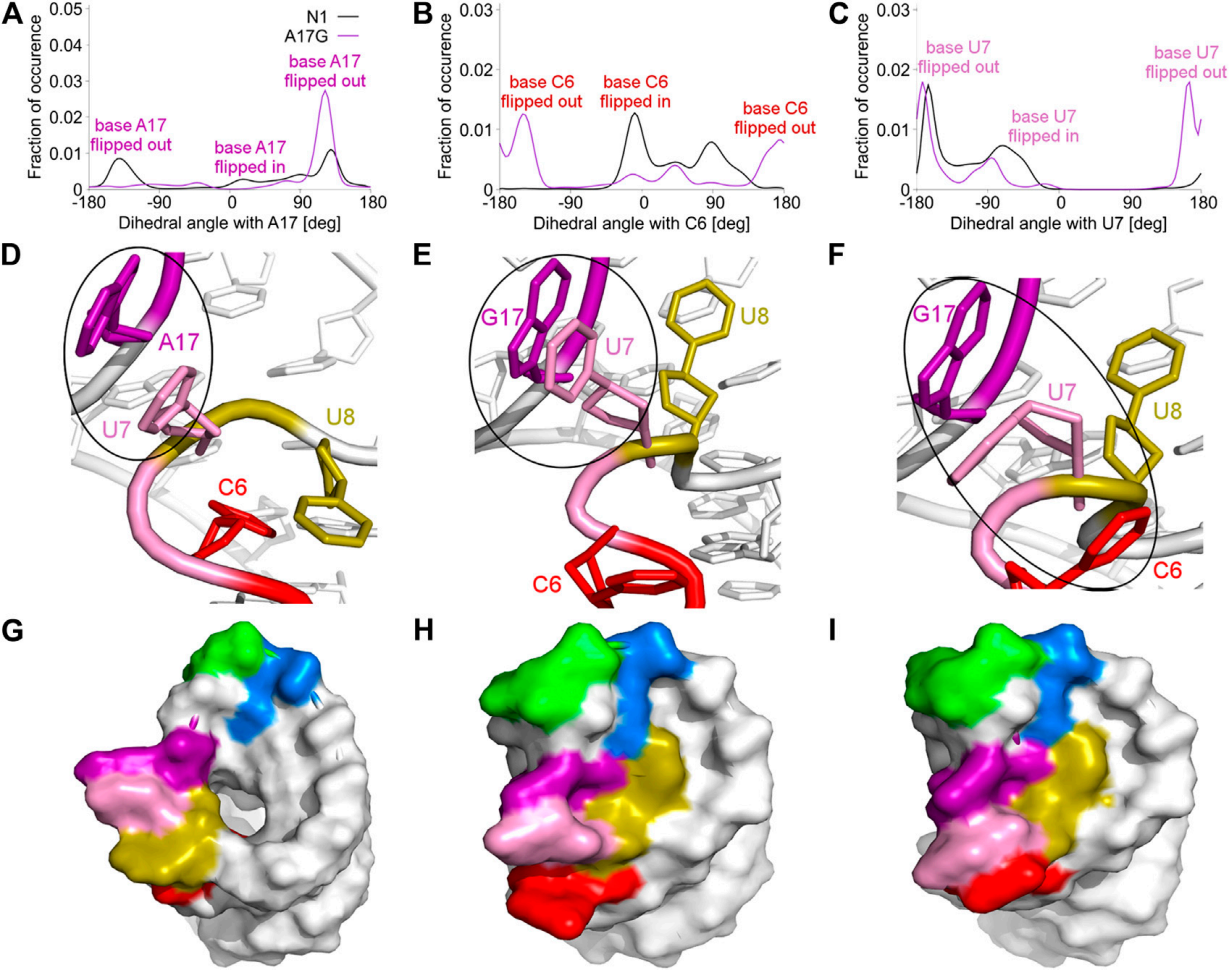
Pseudo-dihedral angles and types of paperclips observed in the unbound N1 and A17G riboswitches. **(A–C)** The distribution of pseudo-dihedral angles for A17, C6 and U7 bases. **(D)** The structure of the paperclip U7:A17 contact in N1 riboswitch. **(E, F)** Structures of paperclips U7:G17 and C6:U7:G17 in the A17G mutant. **(G–I)** Corresponding molecular surfaces.

The A17G mutation changes the dynamics of the C6, U7, U8 and G17 bases, which are able to flip in or out from the riboswitch. A good way to quantify the flipped-in and flipped-out states is to analyze pseudo-dihedral angles formed by selected bases and phosphate groups, which can be carried out according to the scheme proposed by Song *et al.* (Song et al., 2009). The definition of an exemplary pseudo-dihedral angle is shown in Supplementary Figure S11. Of a particular interest are the high peaks for the A17G system at 123° for the A17 base and at 170° for the U7 base, shown in Figure 4A,C, which correspond to the paperclip U7:G17 arrangement. Figure 4D,E presents the structural view of the latter paperclip in N1 and A17G systems. The results of the pseudo-dihedral angle analysis for all systems are shown in Supplementary Figures S12, S13. Stable conformations of C6 and A17 bases in the paperclip C6:A17 in the riboswitches with neomycin correspond to the high peaks in their pseudo-dihedral angle distribution (Supplementary Figure S13). In contrast, the flexible bases in the unbound state reveal a wide distribution of pseudo-dihedral angles.

### 3.4 The U8 base is directed towards the binding site in the unbound A17G riboswitch

**FIGURE 5:**
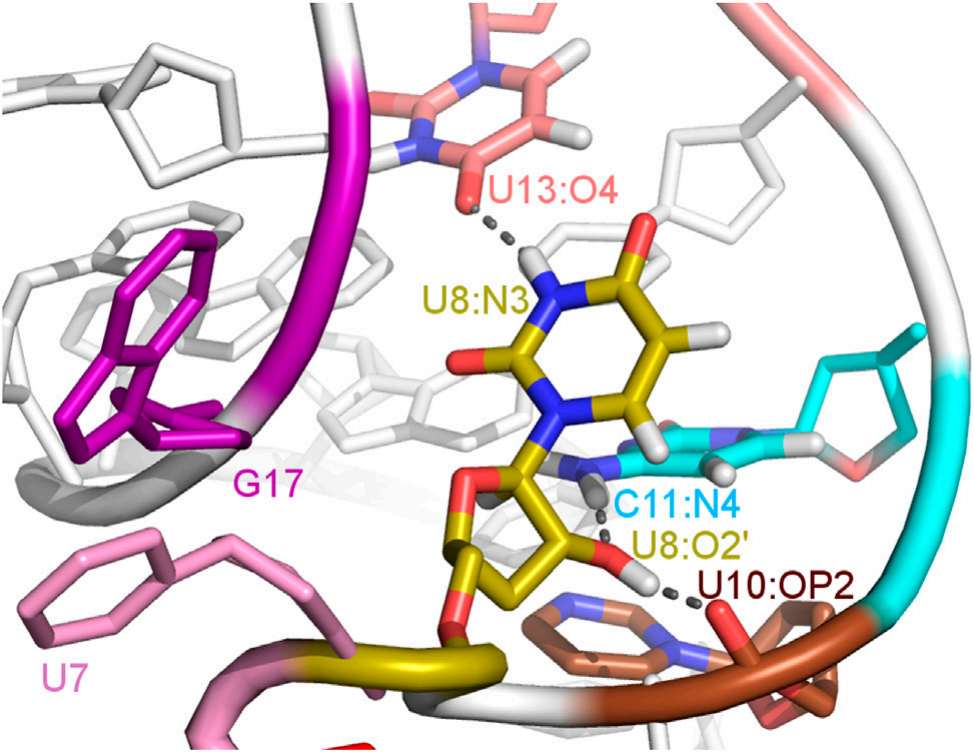
The U8 nucleotide surrounding in the unbound state of the A17G system with hydrogen bonds marked as dashed lines.

The paperclip U7:A17 conformation in the N1 riboswitch is associated with the C6 pseudo-dihedral angle of −9° (Figure 4B). In this conformation, the C6 base is directed towards the riboswitch and, at the same time, U8 is directed outside. Paperclip U7:G17 in the A17G mutant is also described by the U7 pseudo-dihedral angle at 170°, but the C6 and U8 bases locate differently as compared to N1, i.e., C6 is directed outside and U8 inside the riboswitch (Figure 4E). In turn, the structure described by the dihedral angle of -149° for C6, shows a conformation that occurs only in A17G and is characterized by the presence of C6:U7:G17 triple stacking, as shown in Figure 4F. The U8 base acquires the same position as in paperclip U7:G17, namely inside the binding site. Molecular surfaces of the paperclip structures show that the conformation of the U8 base differs between the N1 and A17G systems. In the N1 riboswitch (Figure 4G), the binding site is accessible, but in the A17G mutant this site is occupied by U8 (Figure 4H,I). In the A17G system, the conformation of the U8 base in the binding site is stabilized by three hydrogen bonds (U8:O2’–U10:OP2, U8:O2’–C11:N4 and U8:N3–U13:O4) shown in Figure 5. The occurrence of those hydrogen bonds varies between 37% and 52% of the simulation time in the A17G system and is negligible in all other systems (Table 2). Such position of U8 in the binding site may withhold neomycin from entering the riboswitch according to the conformational selection mechanism and thus result in the depleted activity of the mutated riboswitch. However, this is just a hypothesis since the presence of U8 in the A17G binding pocket was not investigated experimentally.

### 3.5 The A17G mutant changes the hydrogen bond network around neomycin

**FIGURE 6:**
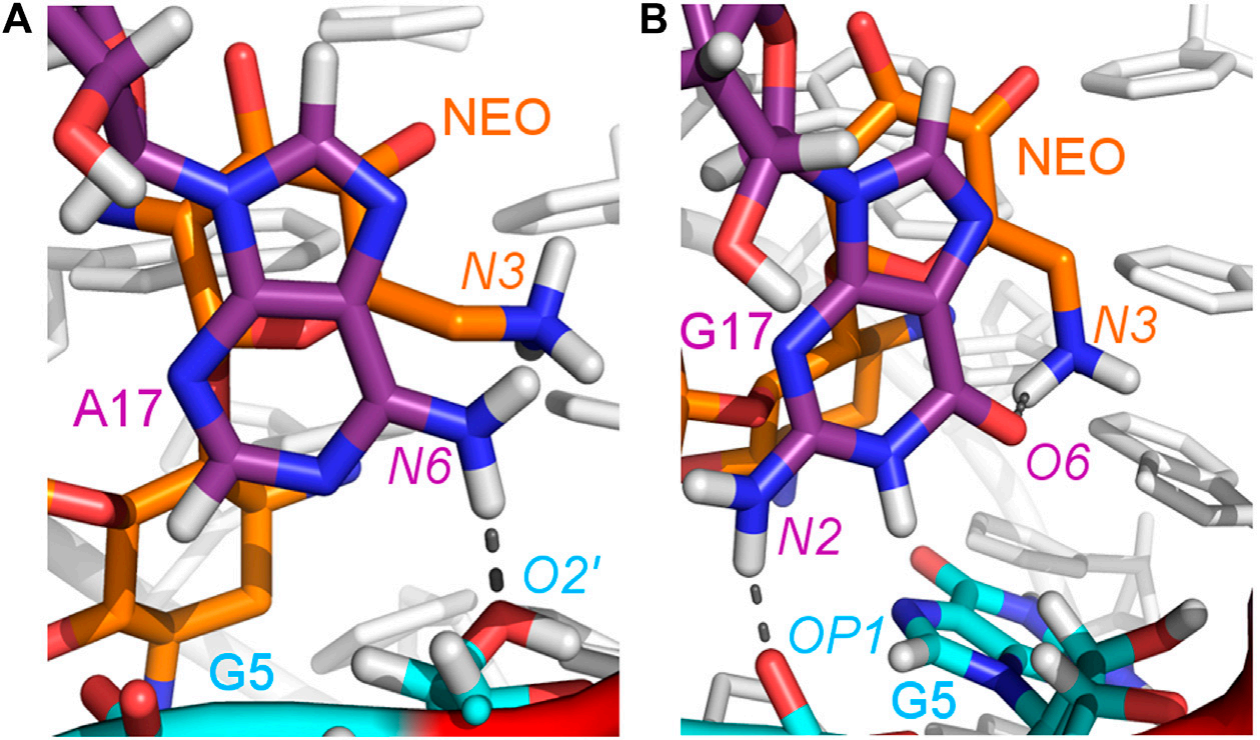
Location of the G5–A17/G17 and G17–N3 hydrogen bonds in: **(A)** N1_NEO and **(B)** A17G_NEO systems.

**FIGURE 7:**
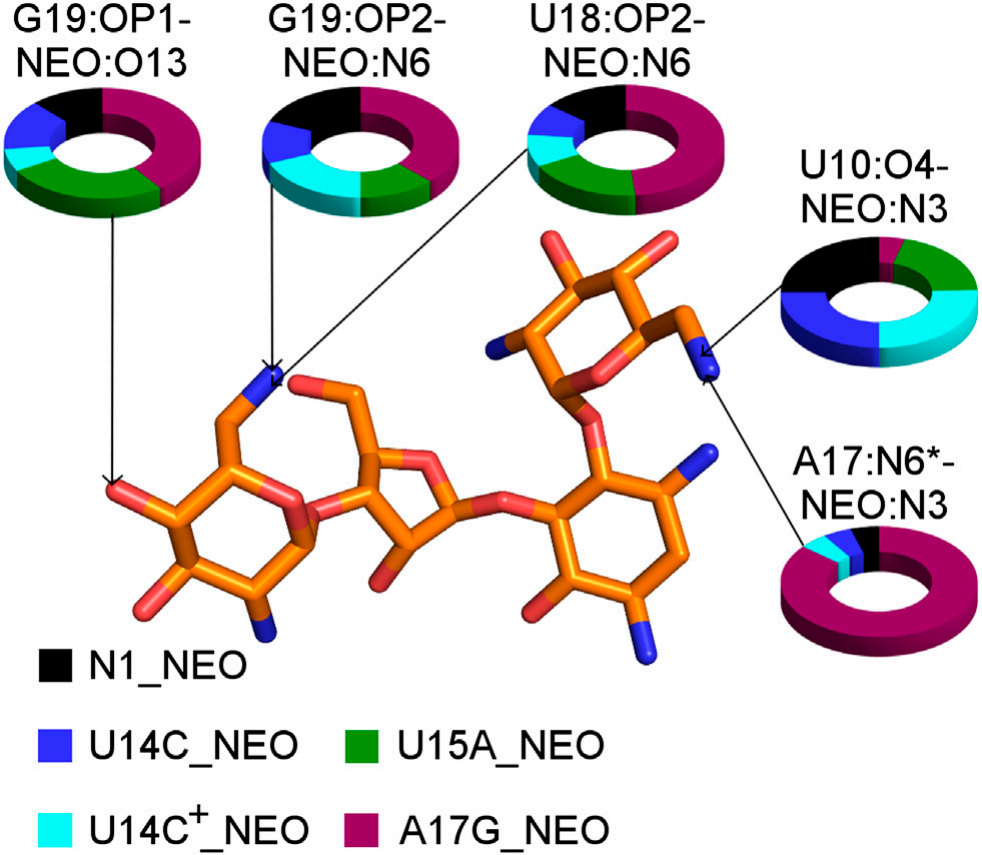
The shares of selected RNA–neomycin interactions. * indicates N6 in A17 and O6 in G17. All RNA–neomycin interactions are listed in Supplementary Table S9.

The A17G mutation changes the interaction network also in the neomycin-bound riboswitch. The A to G substitution breaks the hydrogen bond between G5:O2’ and A17:N6 that occurs in the N1–NEO structure for 70% of the simulation time (Figure 6A and Table 3). In the mutant, the N6 amino group is superseded by a carbonyl group, so a new hydrogen bond is possible with the G5 phosphate oxygen (Figure 6B). However, the latter hydrogen bond is less stable, as shown by hydrogen bond frequency analysis (Table 3). In the N1_NEO, U14C_NEO, U14C^+^_NEO and U15A_NEO systems, the riboswitch interacts with the N3 group of neomycin through a hydrogen bond with U10:O4 for more than half of the simulation time (Figure 7). The amino-to-carbonyl replacement in A17G also results in a new interaction — G17:O6—NEO:N3. This interaction with neomycin stabilizes the G17 base for 55% of the simulation time and is not present in other systems (Supplementary Table S9). The U7 stacking interaction with G17 also becomes possible in the A17G complex with neomycin (Supplementary Table S7). Those interactions point to partial destabilization of G17 in the A17G complex. The RMSF of base 17 in Figure 2B is higher in N1 than in the A17G mutant due to a single event of riboswitch opening in the N1 complex. This is visible as a peak in the RMSD plot between 180 and 200 ns (Supplementary Figure S3B, black line), which reflects an unusual flipped-out position of A17 shown in one of the representative structures of N1 bound state in Figure 3, present in only 7% of conformations. Overall, the RMSD values are on average 0.5 Å higher for the bound A17G structure than for the N1 complex (Supplementary Figure S3). In step with that, neomycin bound to A17G has some additional conformational freedom as its RMSD after RNA fitting is at some points about two times higher than in other complexes (Supplementary Figure S14). Due to different interaction pattern around neomycin, in the A17G complex the ligand is slightly shifted as compared to the complex without mutation (Supplementary Figure S15).

The ITC and fluorescence assays for the N1 and A17G mutant with neomycin are gathered in Table 1. However, the NMR experiments of the riboswitch were conducted with ribostamycin only, due to superior spectral resolution of the ligand resonances and similar binding modes to the riboswitch mutants (Duchardt-Ferner et al., 2010). Neomycin possesses additional ring IV, whose presence decreases the dissociation constant and increases the regulatory activity of N1 riboswitch with respect to ribostamycin (compare the values for the N1 complexes in Table 1). The imino proton solvent exchange rate measurements report that the terminal loop and the U13:U18 base pair in the A17G–ribostamycin complex are less stable than in the N1 complex (Weigand et al., 2014). According to our simulation results, in A17G_NEO, the neomycin ring IV interacts with U18 and G19 phosphate groups. These additional neomycin–RNA hydrogen bonds might contribute to partial U13:U18 base pair stabilization in the A17G complex. As a result, we observe the U13:O2’–U18:N3 hydrogen bond in over 63% and 84% of frames in N1 and A17G complexes, respectively (Supplementary Tables S8, S9). Thus, we anticipate that the simulations of the A17G–ribostamycin complex would lack the stabilizing interactions between RNA and ring IV of the ligand, leading to the lower stability of ribostamycin in the complex, larger dissociation constant and lower regulatory activity in comparison to the A17G–neomycin complex.

Furthermore, the NOESY spectrum of the ^13^C-guanine-labeled RNA suggests the *syn* conformation of G17 in the A17G complex with ribostamycin, in contrast to the *anti* conformation of A17 in the N1–ribostamycin complex (Weigand et al., 2014). In our simulations of the unbound A17G mutant the distribution of G17 χ torsion angles shows the *syn* conformation, while the other unbound riboswitch variants sample the *anti* conformation (Supplementary Figure S16). However, in the complexes with neomycin, all the systems prefer the *anti* conformation, with a slight shift in the orientation of the G17 base position due to a different interaction network as pointed out earlier. The sampling of the *syn* conformation by G17 seen in NMR experiments led the authors to suggest that the N3 group of ribostamycin ring I of ribostamycin does not interact with G17, the C6:G17 stacking interaction is prevented, which increases ligand flexibility (Weigand et al., 2014). Taken together, our results point to other reasons for the observed higher flexibility of the ligand in the simulations; the C6:G17 stacking is present and a strong hydrogen bond between G17:O6 and NEO:N3 directs neomycin to bind in a slightly shifted and less stable position in the A17G binding site with respect to the N1 complex (Supplementary Figure S15).

### 3.6 The gREST simulations suggest the protonation of cytosine 14 in the U14C mutant

**FIGURE 8:**
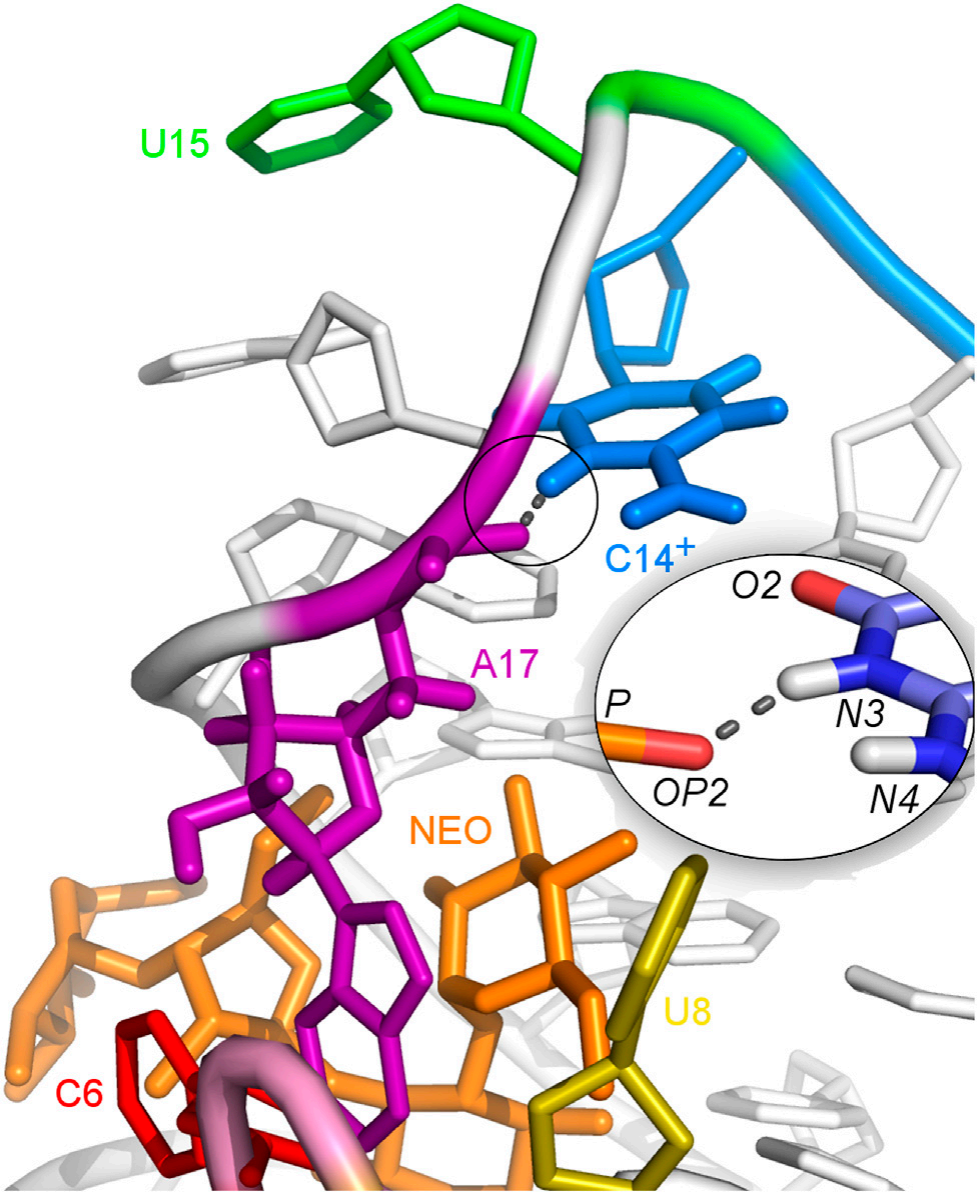
Hydrogen bonds between C14^+^ and A17 observed in the U14C^+^_NEO system. The proton connected to the N3 atom is missing in the U14C mutant without cytosine protonation.

The U-turn motif is characterized by the presence of these hydrogen bonds U14:O2’–A16:N7, U14:N3–A17:P and A16:O2’–U18:P (Weigand et al., 2014). Two of these hydrogen bonds, between U14–A16 and A16–U18 are present in all the simulations of the neomycin–riboswitch complexes. In the U14C complex without protonated C, the Watson-Crick edge lacks the proton underlying the C14:N3–A17:P hydrogen bond. Therefore, in Table 3, the C14:N3–A17:OP2 hydrogen bond in the U14C_NEO system is absent and there are no other C14–A17 hydrogen bonds, which destabilizes the U-turn. On the other hand, the protonated cytosine can form C14^+^:N3–A17:OP2 interaction, shown in Figure 8, and restore the stability of the U-turn. This hydrogen bond is present for 67% of the simulation time in the U14C^+^_NEO system, which is close to 70% of simulation frames for the hydrogen bond with U14 in N1_NEO (Table 3). The U to C function-retaining replacements in the U-turn motifs have been reported previously and confirmed in the context of the N1 riboswitch (Gottstein-Schmidtke et al., 2014; Krepl et al., 2018).

In the unbound state of the riboswitch, the above-mentioned hydrogen bonds that form the U-turn are rare, even in the U14C^+^ case (Supplementary Table S6). Thus, when no ligand is present, the protonation of C14 is not enough for the U-turn formation. Lack of U-turn causes large fluctuations of the apical loop in all systems (Figure 2A), especially in the U14C system, while A17 in the U14C^+^ mutant fluctuates less than in other systems. Also, the χ torsion angles of A17 in the U14C^+^ mutant sample the *syn* conformation, similarly to the A17G system, while the N1 riboswitch samples the *anti* conformation. In the unbound riboswitches, the highest peaks in the pseudo-dihedral angle distributions of C6, U7, U8, and A17 bases observed in the N1 system coincide more often with the highest peaks in the U14C than in the U14C^+^ system (Supplementary Figure S12). Those deviations of the U14C^+^ system from the N1 system prove that the protonation of C14 cannot mimic the interactions and dynamics of U14 in the N1 riboswitch. This means that the U14C^+^ system is closer to inactive A17G mutant, which may significantly reduce the number of conformations preformed for ligand binding. According to the experimental data, the U14C mutation reduces the N1 riboswitch activity 3-fold, but it is not as small as for the A17G system (Table 1). Also, the dissociation constant of the neomycin -U14C mutant is 3 times larger than for the N1 system but almost 6 times smaller than for the A17G system. Thus, the cytosine in the U14C mutant in the unbound state is probably protonated to a low extent, which would agree with the imino proton NMR signals for the U14C mutant (Weigand et al., 2014).

### 3.7 The U15A mutant displays conformations preformed for ligand binding

Mutating U15 to A only slightly affects the dynamics of the riboswitch, which is visible in the similar fluctuations in the unbound and bound states of the N1 and U15A variants in Figure 2. The only difference with respect to the unmutated structure is the presence of the bound state conformations in the unbound state, such as the paperclip C6:A17 that appears in the second most populated cluster in the unbound U15A trajectory ‐ Cluster 2 in Supplementary Figure S8A. The stacking interaction C6:A17 is not visible in the N1 simulation (Table 2). Also, the pseudo-dihedral angle distributions of C6, U8 and A17 in the unbound state resemble those from the complex (Supplementary Figures S12, S13). Comparison of 1D imino-proton NMR signals of the ligand-free N1 and U15A systems indicates stronger signals for the U13/18 and U10/21 resonances in the U15A system (Weigand et al., 2014), confirming the higher degree of preformation in the latter system. This is in agreement with the frequency of U13:O2—U18:N3 and U10:O2—U21:N3 hydrogen bonds in our simulations, which is more than 15% higher in the U15A simulations than in N1 simulations (Supplementary Table S6). Thus, the U15A mutation facilitates the conformational selection and, as a result, increases the regulatory activity of the N1 riboswitch toward neomycin as evidenced by the fluorescence assay (Weigand et al., 2014).

## 4 Conclusion and Outlook

We performed the gREST simulations for a set of single-point N1 riboswitch mutants in the unbound and neomycin-bound states. The dynamics of the interactions unveiled the reasons behind the hindered activity of the A17G mutant. The A17G mutation affects the unbound riboswitch dynamics because additional bulge-apical loop contacts are formed that act as paperclips and narrow the set of conformations necessary for neomycin entering the riboswitch. This is in line with the conformational selection mechanism suggested for the N1 riboswitch. Furthermore, the dynamical studies of the U14C mutant in different protonation states of C14 support the protonated state of this cytosine in the bound state and deprotonated one in the unbound state. Additionally, the dynamics of the U15A mutant resembles the dynamics of the riboswitch without mutation, except that in the unbound state the U15A mutant displays more conformations preformed for ligand binding than N1. This corresponds to the slightly higher regulatory activity of this mutant compared to the unmutated riboswitch (Weigand et al., 2014). Overall, the U15A mutant is the most effective among the studied riboswitch sequences because the dynamics of this mutant in the unbound state facilitates conformational selection for neomycin binding. Our studies of the mutations introduced to the N1 apical loop corroborated the experimental data and provided insight into the riboswitch dynamics–activity relationship. In the future it would be interesting to investigate the conformational dynamics of N1 replacing also the bulge nucleobases, which could unveil the mutations compensating the apical loop ones analyzed in this work.

## Data Availability

The raw data supporting the conclusions of this article will be made available by the authors, without undue reservation.
