# Supplementary material for: Mutations of N1 Riboswitch Affect its Dynamics and Recognition by Neomycin Through Conformational Selection
Source: Front Mol Biosci. 2021 Feb 18;8:633130. doi: 10.3389/fmolb.2021.633130 (PMC7942488; doi:10.3389/fmolb.2021.633130)
Supplement: Supplementary file 1 [file datasheet1.pdf]

Supplementary Material

Mutations of N1 Riboswitch Affect its Dynamics and Recognition by Neomycin Through Conformational Selection

Piotr Chyży [1,2], Marta Kulik [1,3,*], Suyong Re [4,5], Yuji Sugita [4,6,7] and Joanna Trylska [1]

[1]Centre of New Technologies, University of Warsaw, Warsaw, Poland

[2]Faculty of Physics, University of Warsaw, Warsaw, Poland

[3]Biological and Chemical Research Centre, Department of Chemistry, University of Warsaw, Warsaw, Poland

[4]RIKEN Center for Biosystems Dynamics Research (BDR), Kobe, Japan

[5]National Institutes of Biomedical Innovation, Health and Nutrition, Ibaraki, Japan

[6]RIKEN Cluster for Pioneering Research (CPR), Wako, Japan

[7]RIKEN Center for Computational Science, Kobe, Japan

Correspondence*: m.kulik@uw.edu.pl

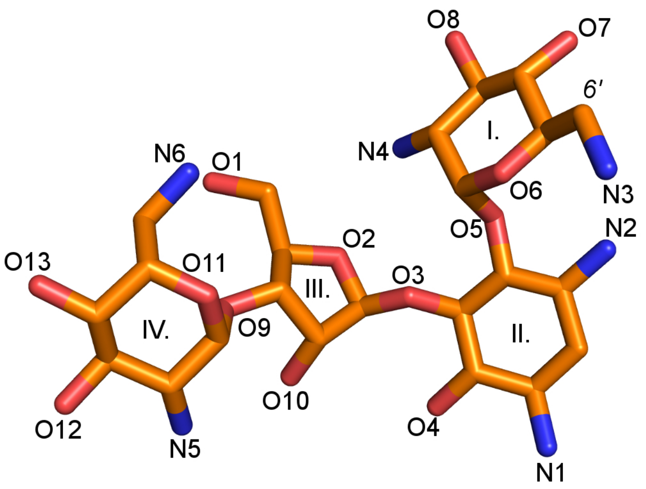

Figure S1. The structure of neomycin. The numbering of the rings is shown inside the rings. Atom names are given according to the Amber naming scheme. The highlighted 6′-carbon atom indicates the position of the functional group that was modified to obtain neomycin from paromomycin.

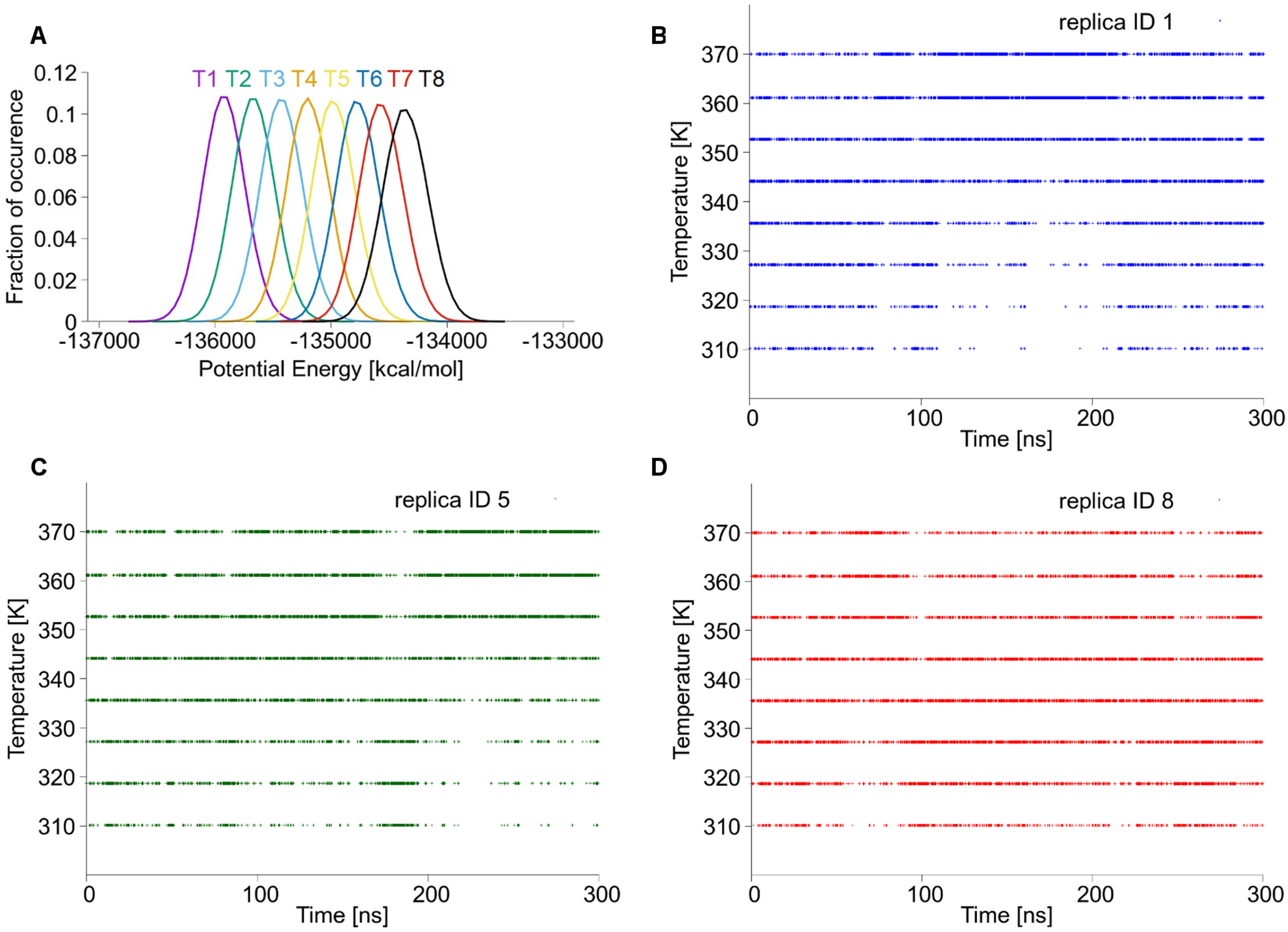

Figure S2. Sampling in the gREST simulation of the N1_NEO riboswitch. (A) Distribution of the potential energy of the solute at different temperatures T1, T2, ..., T8. The random walk in the temperature space shown for replica 1 (B), replica 5 (C) and replica 8 (D).

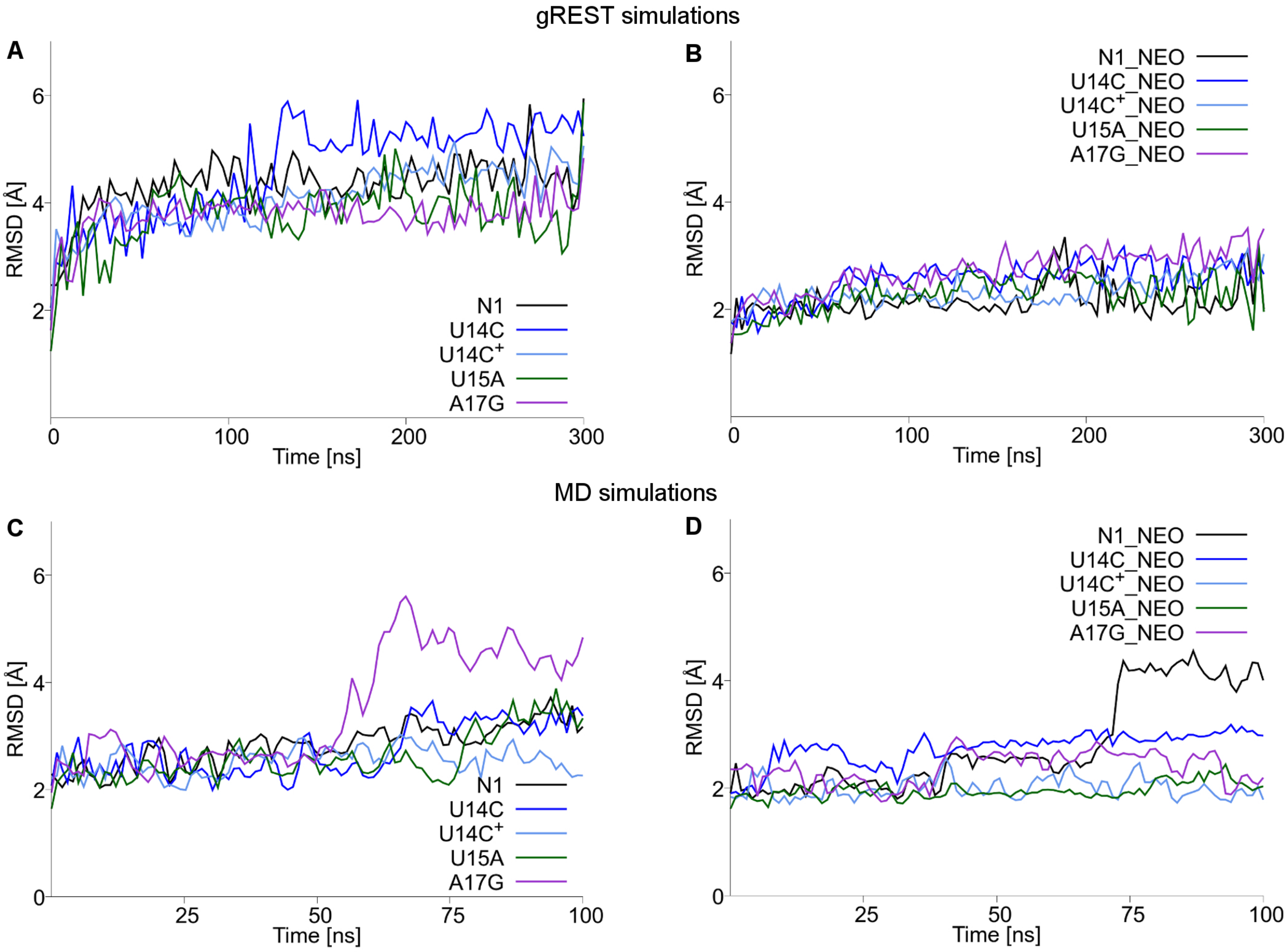

Figure S3. RMSD values for the RNA non-hydrogen atoms of the unbound (A) and bound (B) systems in gREST and the unbound (C) and bound (D) systems in MD. The gaussian_filter1d method was used to smooth the data.

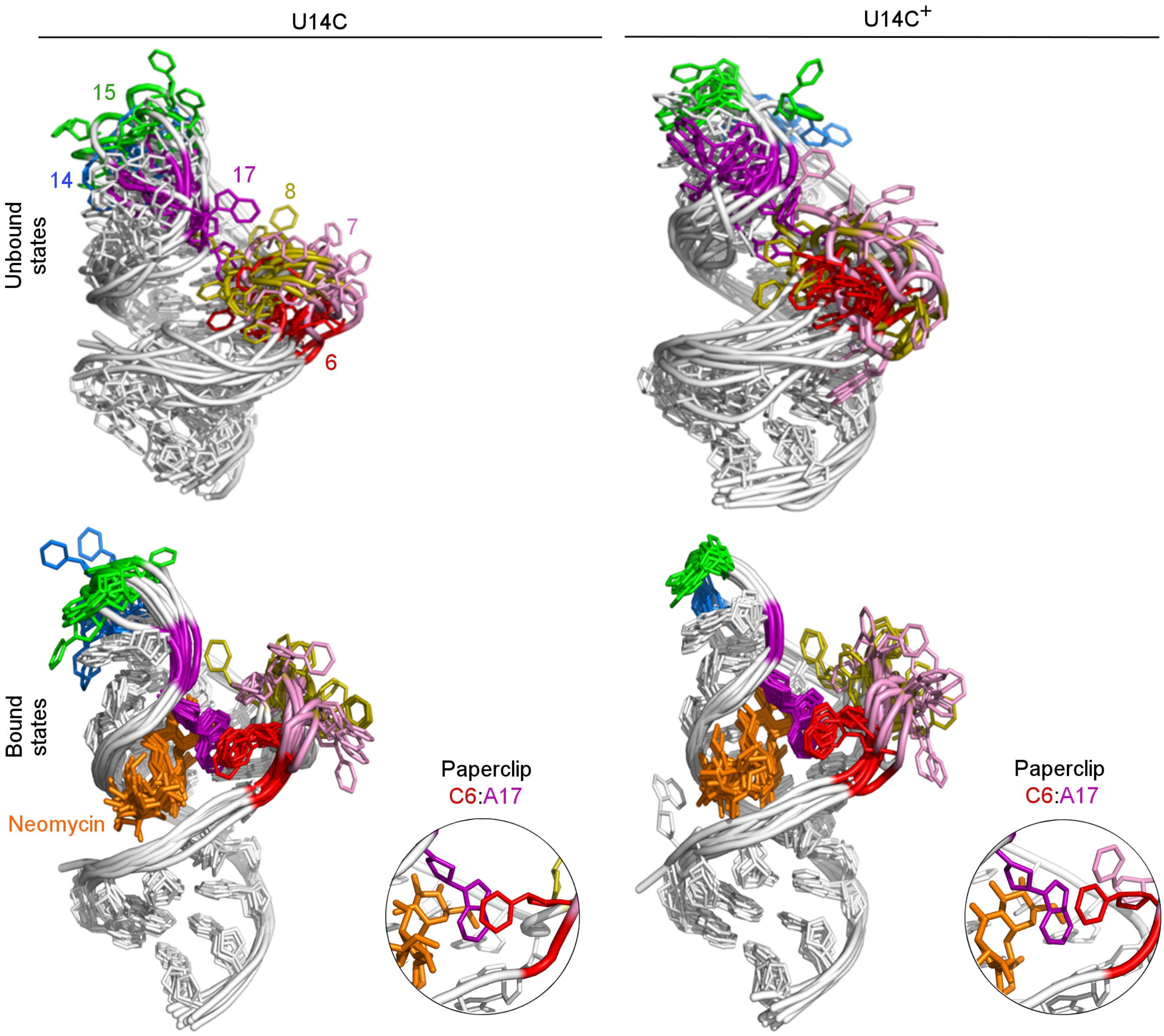

Figure S4. Superposition with respect to phosphorus atoms of 10 representative structures from clustering for U14C and U14C$^+$ simulations. The paperclips – the Van der Waals interactions between the bulge and apical loop seen in at least 25% of simulation frames – are shown in the insets for cluster representants.

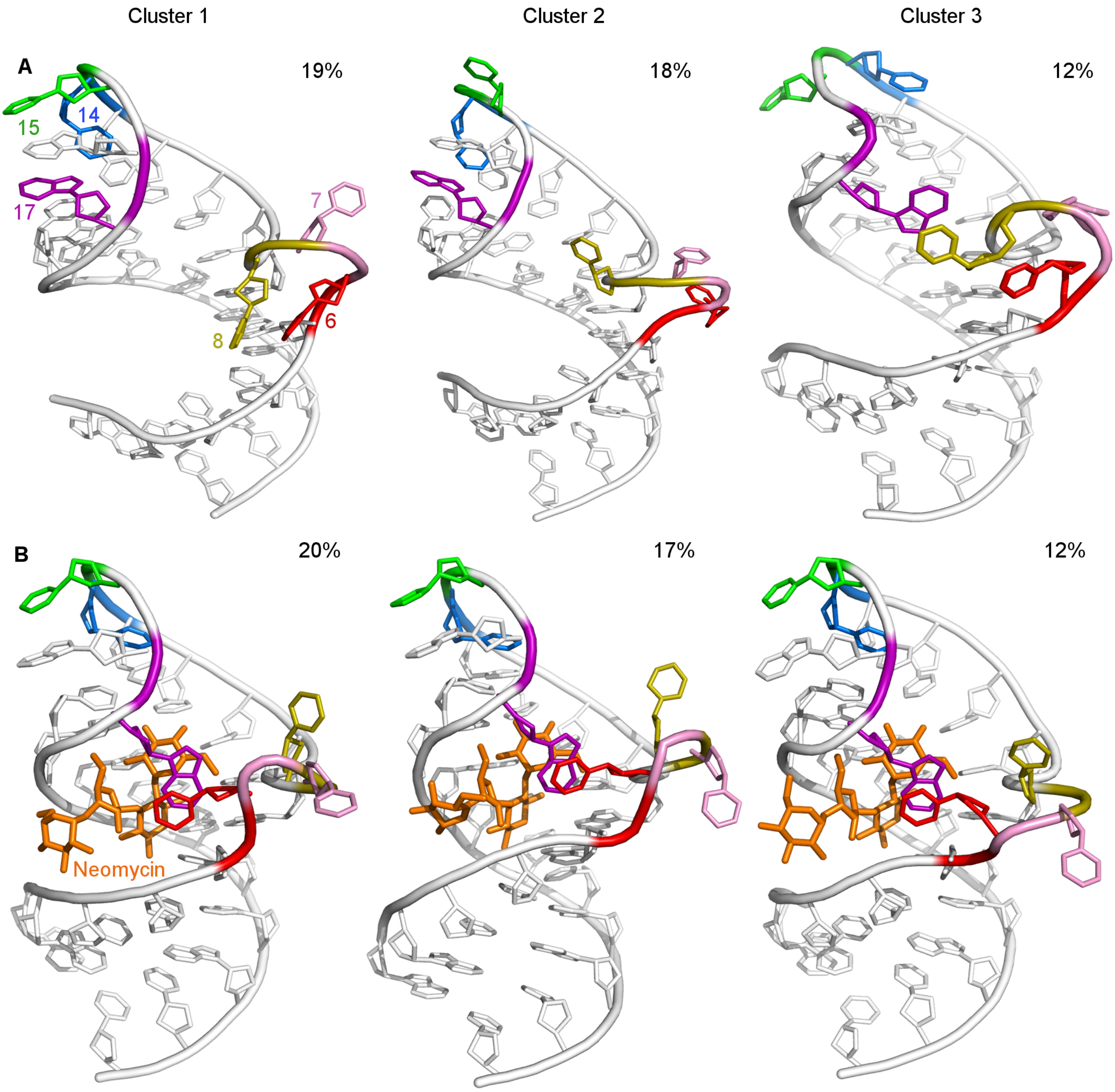

Figure S5. The representative structures of three most populated structural clusters in the N1 riboswitch: (A) unbound state, (B) bound state. Next to each structure, the percentage of occurrence in the analyzed trajectory is shown.

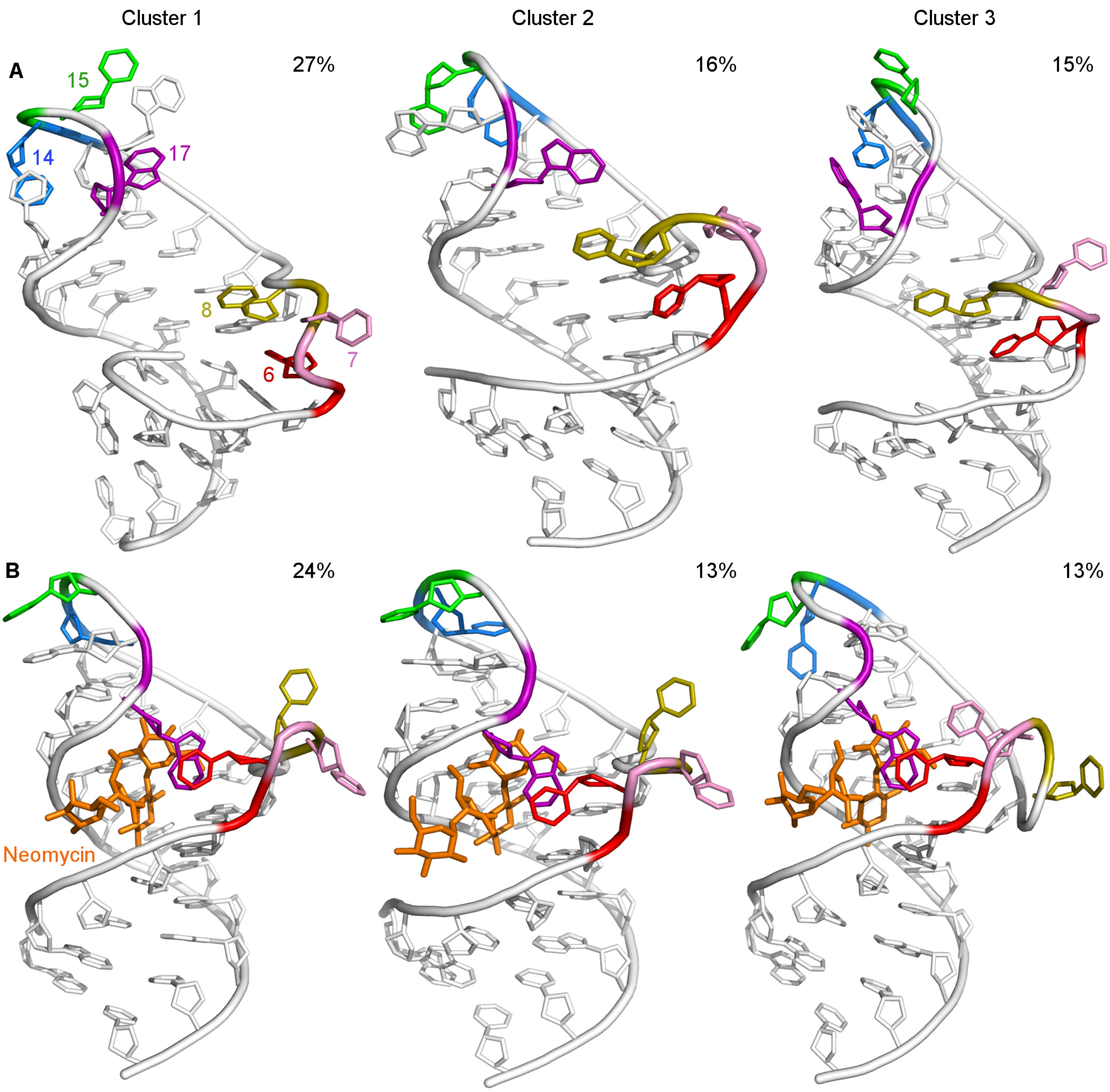

Figure S6. The representative structures of three most populated structural clusters in the U14C riboswitch: (A) unbound state, (B) bound state. Next to each structure, the percentage of occurrence in the analyzed trajectory is shown.

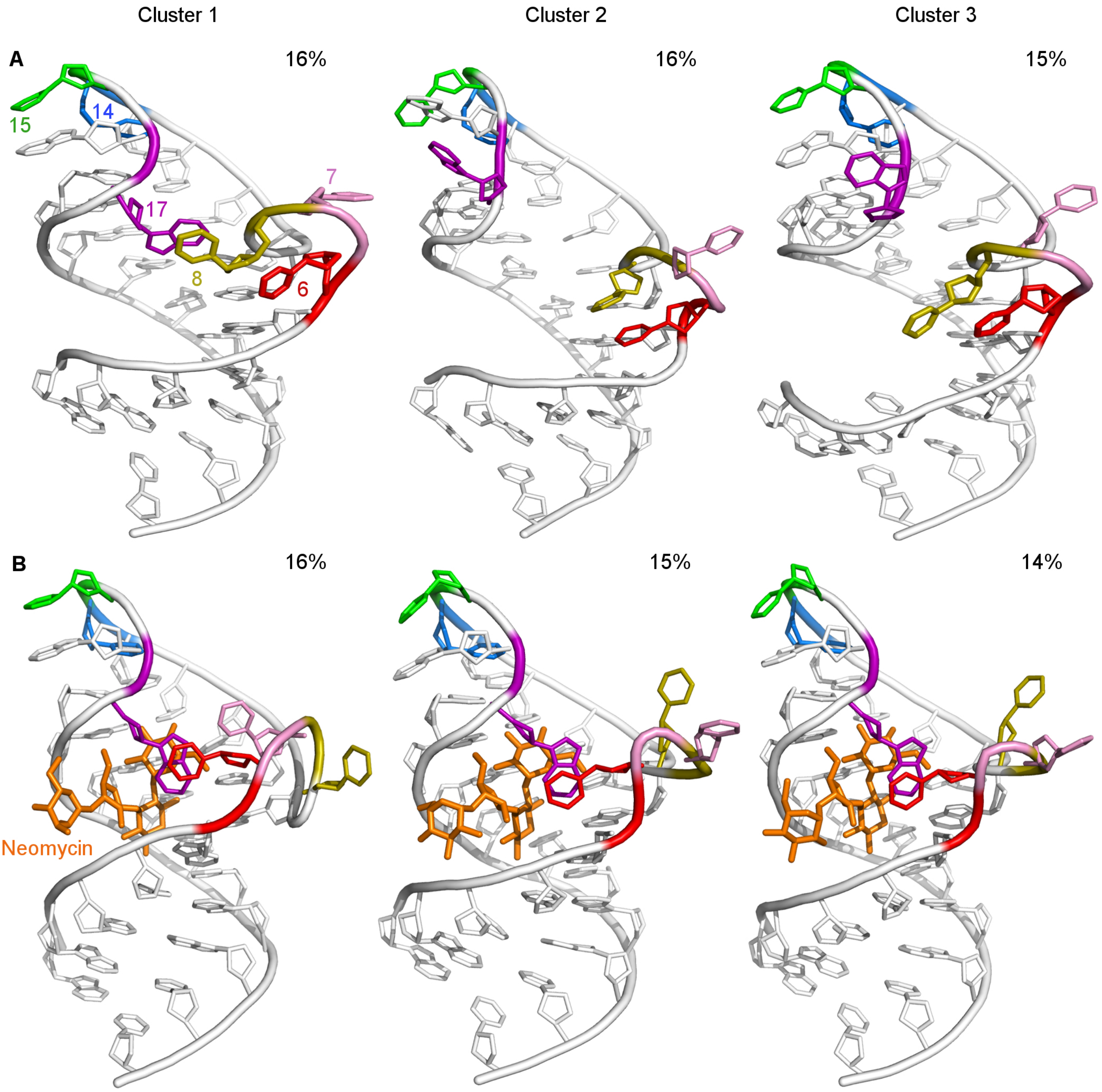

Figure S7. The representative structures of three most populated structural clusters in the U14C$^+$ riboswitch: (A) unbound state, (B) bound state. Next to each structure, the percentage of occurrence in the analyzed trajectory is shown.

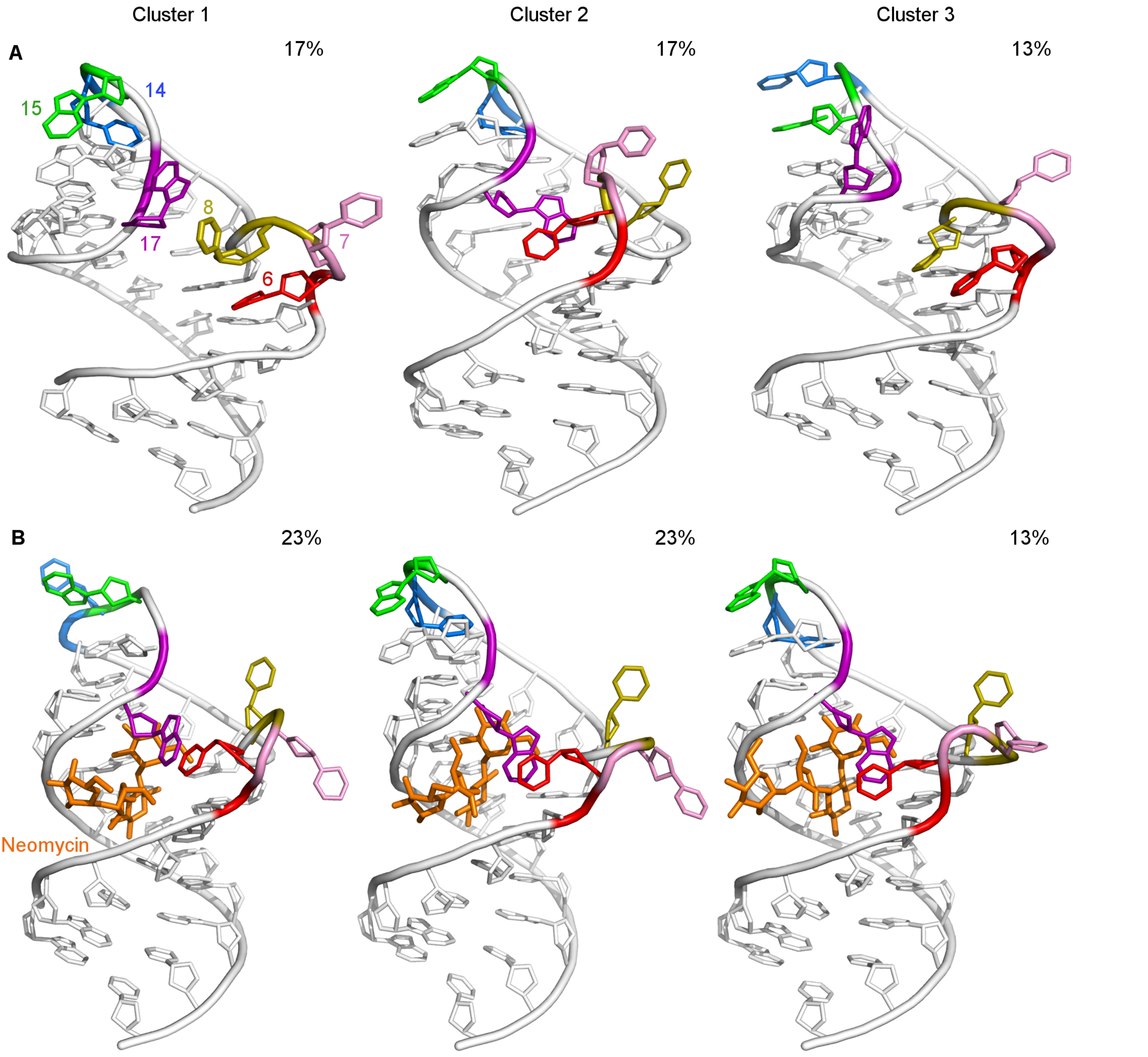

Figure S8. The representative structures of three most populated structural clusters in the U15A riboswitch: (A) unbound state, (B) bound state. Next to each structure, the percentage of occurrence in the analyzed trajectory is shown.

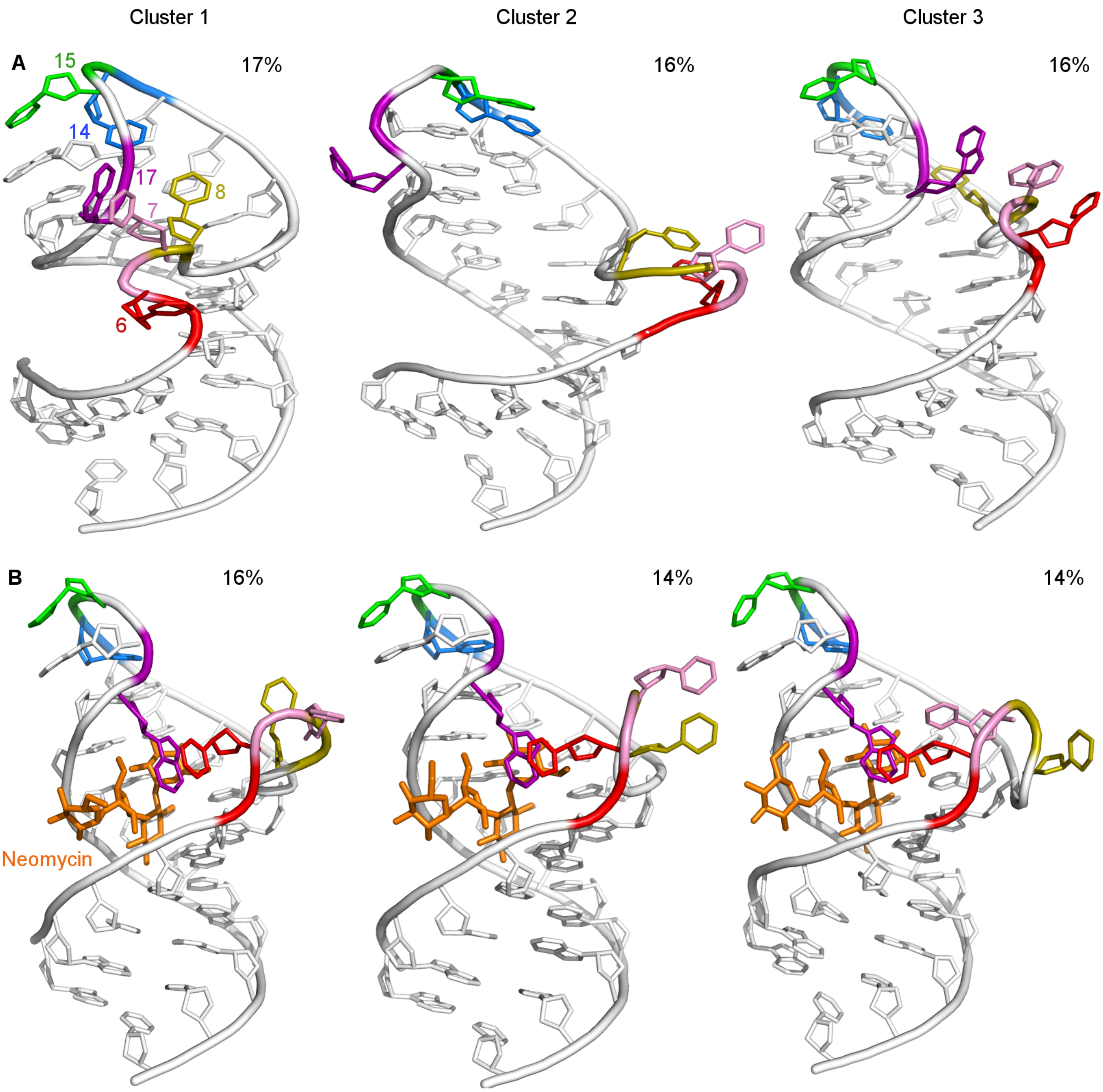

Figure S9. The representative structures of three most populated structural clusters in the A17G riboswitch: (A) unbound state, (B) bound state. Next to each structure, the percentage of occurrence in the analyzed trajectory is shown.

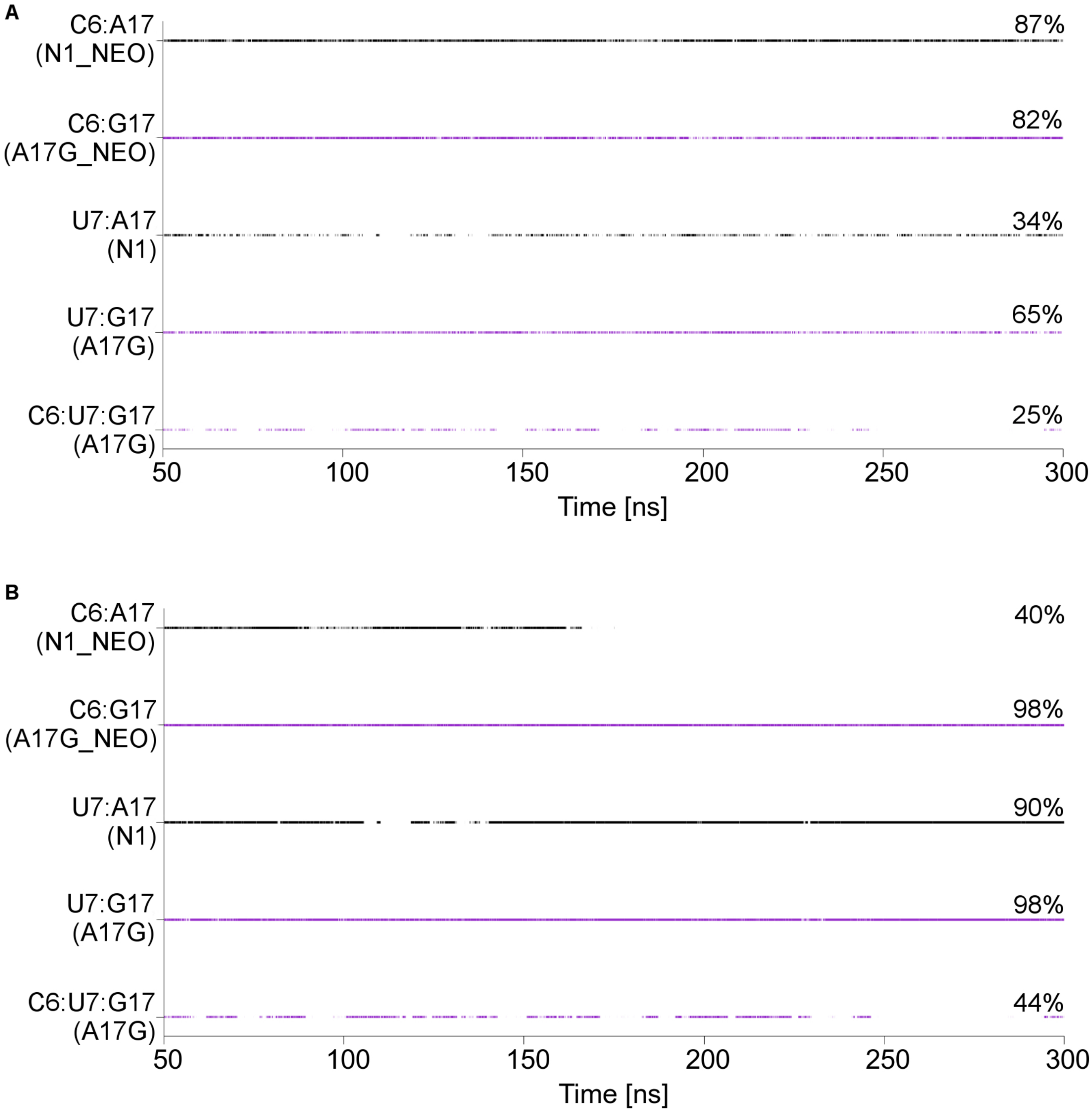

Figure S10. The paperclip interactions created and broken in the time course of the N1 and A17G trajectories, calculated for the gREST trajectories at 310 K (A) and trajectories of the replica ID 1, visiting different temperatures (B).

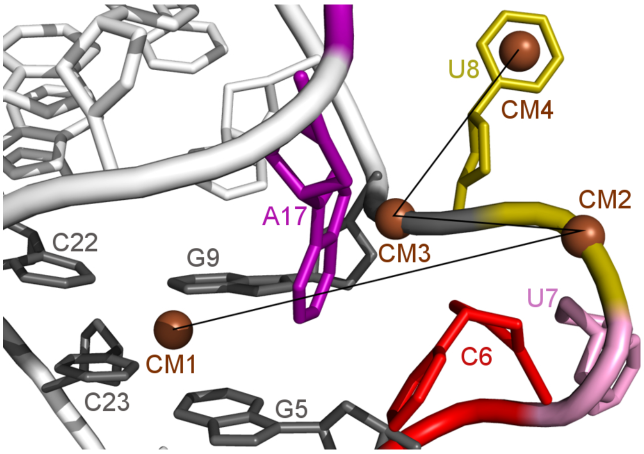

Figure S11. Definition of the pseudo-dihedral angle calculated to assess the flipping of bases. The dihedral angle was measured using four centers of masses, represented using configuration for U8 calculations. CM1 defines the center of mass of G5, G9, C22, C23 nucleobases, CM2 and CM3 are calculated for U8 and G9 phosphate group atoms, CM4 reflects U8 nucleobase center of mass.

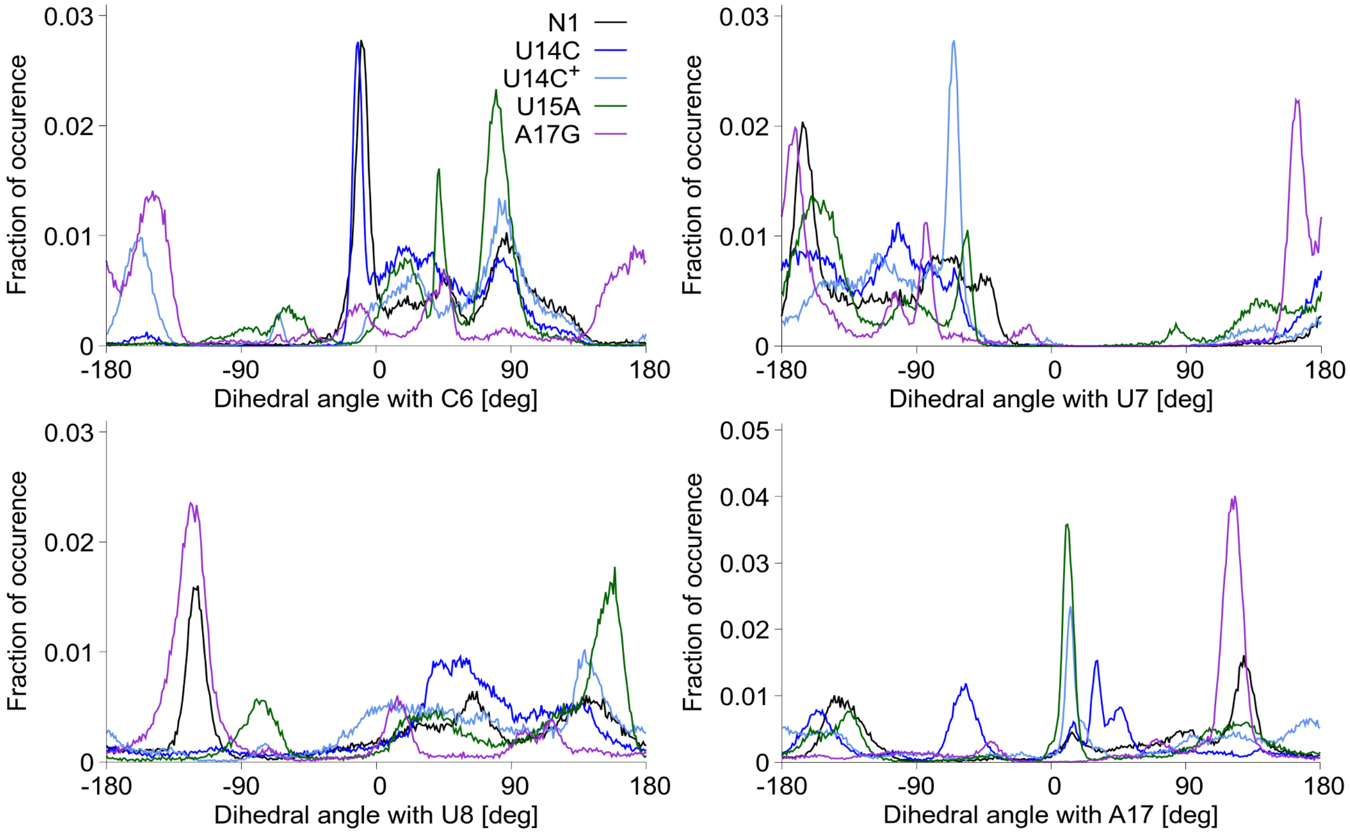

Figure S12. The distribution of pseudo-dihedral angles showing the flipping of nucleotides C6, U7, U8 and A17 in the unbound systems.

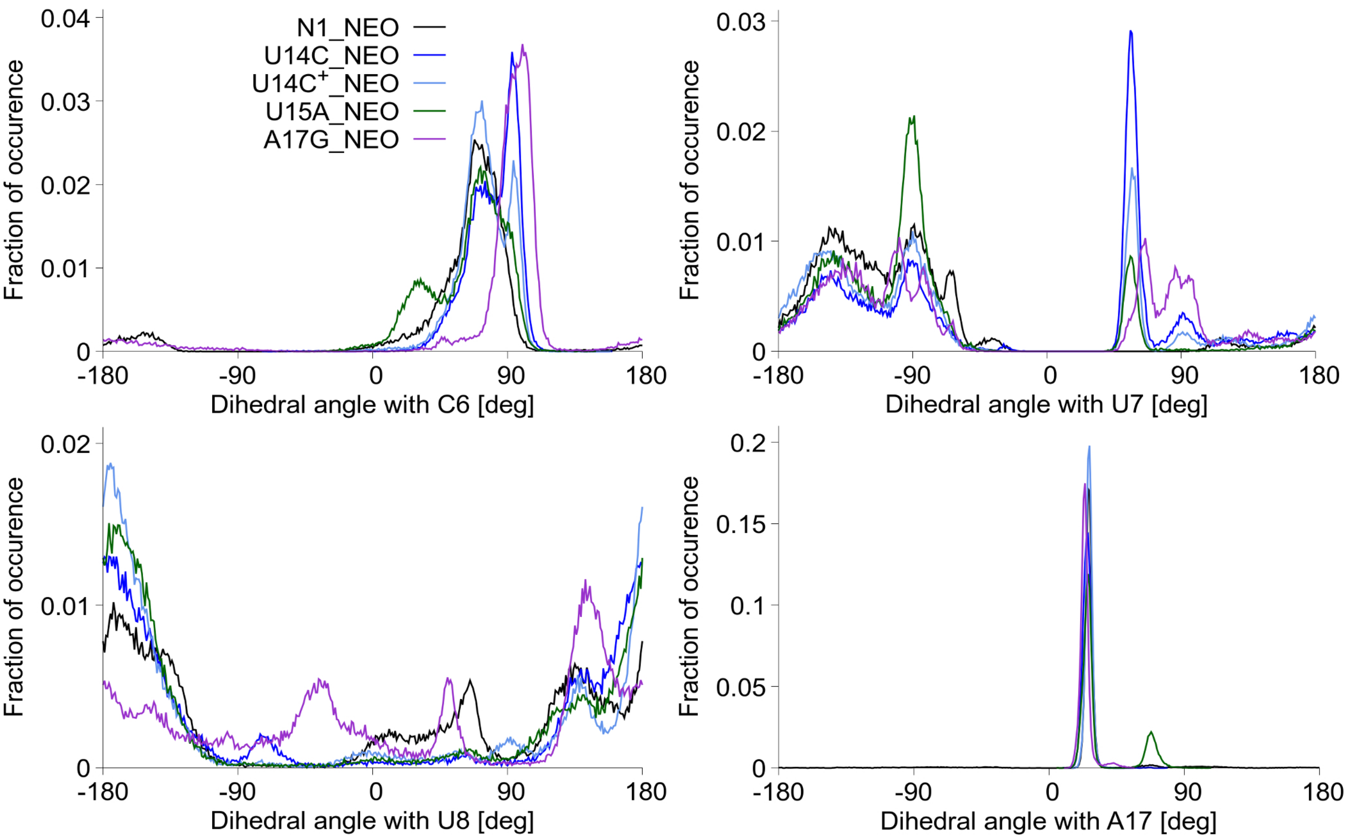

Figure S13. The distribution of dihedral angles showing the flipping of nucleotides C6, U7, U8 and A17 in the bound systems.

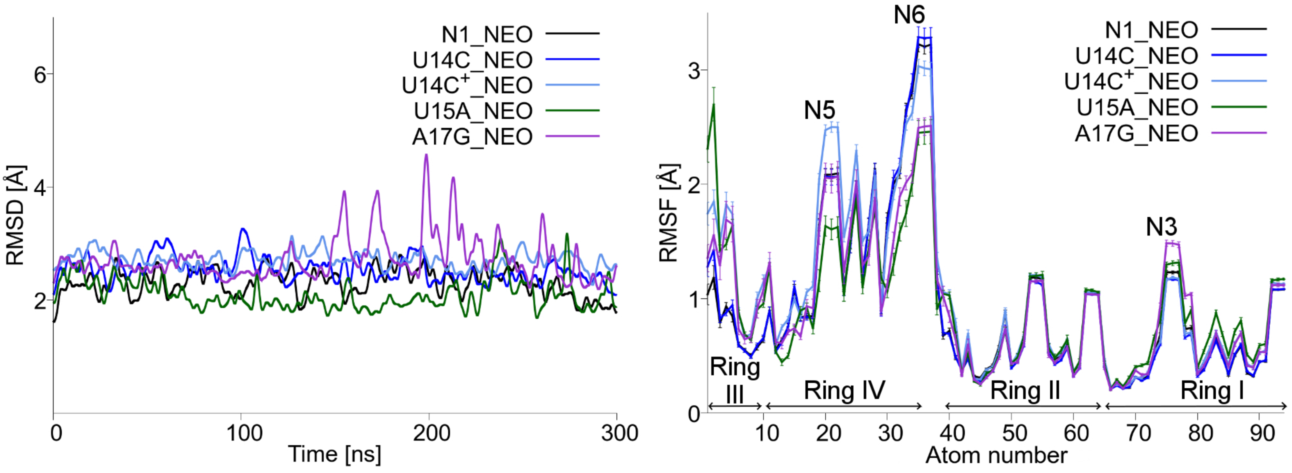

Figure S14. RMSD values for non-hydrogen atoms of neomycin after superimposing the RNA non-hydrogen atoms on the reference initial structure (left) and RMSF for the all atoms of neomycin with respect to the average structure. The gaussian_filter1d method was used to smooth the data for RMSD calculations.

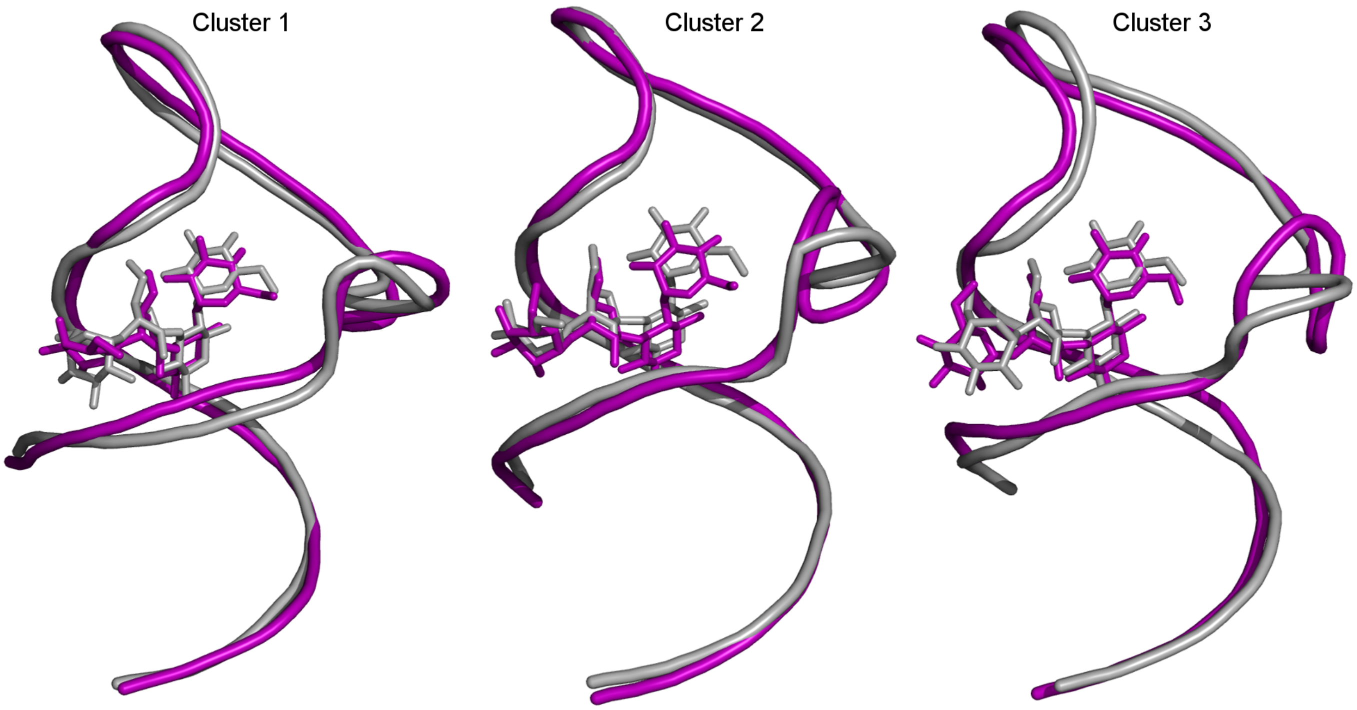

Figure S15. Three highly populated clusters from N1_NEO (in gray) and A17G_NEO (in purple) simulations with neomycin superimposed by phosphorus atoms.

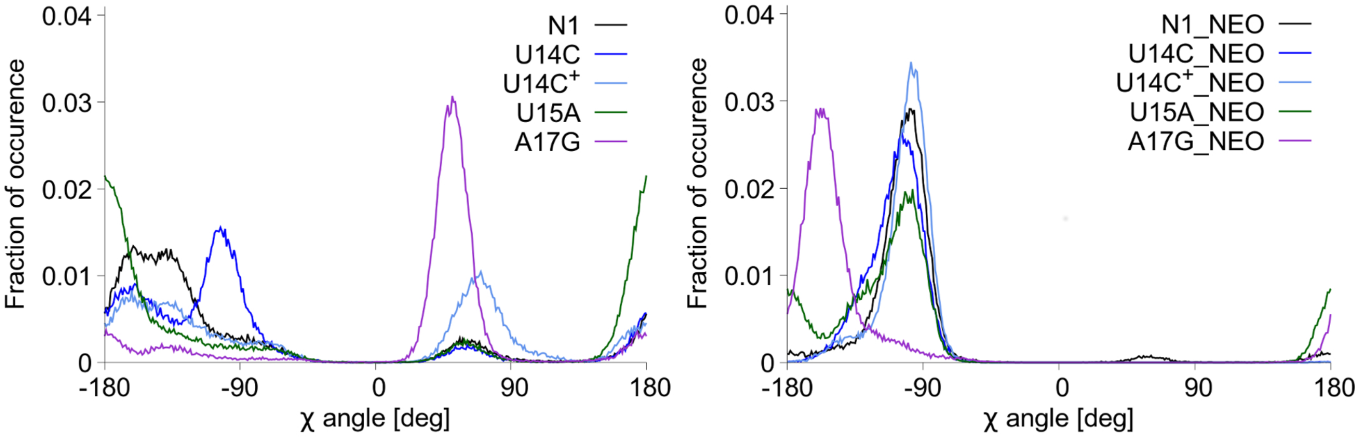

Figure S16. The χ torsion angle distributions for A17/G17 in unbound (left) and bound (right) systems. The A17/G17 bases that sample the negative χ torsion angles acquire the *anti* conformation and the positive angles denote the *syn* conformation.

Table S1. Simulations performed for the N1 riboswitch and its mutants in the NVT ensemble.

System	MD	gREST
N1	100 ns, 310.15 K	8 replicas
U14C		300 ns each, 310.15 – 370.00 K
U14C$^+$		Solute region: RNA + Counterions
U15A		Solute energy terms: Dihedral, Coulomb, LJ
A17G		
N1_NEO	100 ns, 310.15 K	8 replicas
U14C_NEO		300 ns each, 310.15 – 370.00 K
U14C$^+$_NEO		Solute region: RNA + Counterions + NEO
U15A_NEO		Solute energy terms: Dihedral, Coulomb, LJ
A17G_NEO		

Table S2. Overlap between the covariant matrices of the final fragments of the gREST trajectories: 250 – 275 ns and 275 – 300 ns, calculated with the Gromacs gmx anaeig tool. For the formulas for the normalized and shape overlaps, see the gmx anaeig manual.

System	Normalized overlap	Shape overlap
N1	0.66	0.66
U14C	0.62	0.62
U14C$^+$	0.60	0.61
U15A	0.60	0.60
A17G	0.70	0.70
N1_NEO	0.65	0.66
U14C_NEO	0.69	0.69
U14C$^+$_NEO	0.72	0.73
U15A_NEO	0.66	0.67
A17G_NEO	0.69	0.69

Table S3. Acceptance ratio of the exchanges between replicas in the gREST simulations.

		Acceptance ratio				
Replica ID	Replica ID	N1	U14C	U14C$^+$	U15A	A17G
1	2	0.19	0.18	0.19	0.19	0.17
2	3	0.20	0.20	0.20	0.19	0.19
3	4	0.22	0.21	0.21	0.21	0.21
4	5	0.23	0.23	0.23	0.22	0.22
5	6	0.24	0.25	0.24	0.23	0.24
6	7	0.25	0.26	0.26	0.24	0.25
7	8	0.24	0.25	0.25	0.25	0.22
Replica ID	Replica ID	N1_NEO	U14C_NEO	U14C$^+$_NEO	U15A_NEO	A17G_NEO
1	2	0.23	0.22	0.23	0.22	0.22
2	3	0.24	0.24	0.24	0.23	0.23
3	4	0.26	0.26	0.26	0.25	0.25
4	5	0.27	0.27	0.28	0.27	0.27
5	6	0.28	0.29	0.29	0.28	0.29
6	7	0.30	0.30	0.31	0.30	0.30
7	8	0.29	0.29	0.30	0.29	0.29

Table S4. Percentage of Van der Waals interactions between two nucleobases in the simulations of riboswitches in the unbound state. The first 50 ns of the simulations were excluded from the analysis. Only the stacking interactions that appear in more than 25% of the analyzed trajectory in at least one system are shown. The following stacking interactions are stable (observed in at least 90% frames of analyzed part of trajectory): G2:C3, C3:U4, U4:G5, U4:G25, G5:G9, G5:A24, G9:U10, G9:C23, U10:C11, U10:C22, C11:C12, C11:U21, C12:G20, G19:G20, G20:U21, U21:C22, C22:C23, C23:A24, A24:G25, G25:U26, U26:C27. The nucleotide sequence is shown in Figure 1.

		Percentage of simulation time [%]				
Base 1	Base 2	N1	U14C	U14C$^+$	U15A	A17G
G1	G2	95	67	94	96	99
G1	C22	0	30	0	0	0
G2	C27	94	73	95	94	98
C3	U26	88	86	87	87	94
U4	C6	13	28	14	25	10
G5	C6	48	56	24	38	16
G5	G25	38	45	41	44	48
C6	U7	11	1	0	1	39
C6	U8	41	49	52	24	14
C6	G9	13	49	14	28	13
C6	A17	0	11	1	34	7
U7	U8	4	4	2	32	17
U7	A17	34	2	10	2	65
U8	C12	0	0	1	6	54
G9	A24	24	20	31	40	39
C12	U13	100	86	100	94	100
U13	U14	93	83	93	73	92
U13	G19	98	77	99	86	99
U14	U15	17	25	12	30	28
U14	A16	47	34	52	40	56
U14	U18	70	38	75	55	62
U14	G19	23	35	7	3	13
U15	A16	68	75	91	88	92
A16	A17	38	57	43	8	3
A16	U18	58	25	56	74	99
A17	U18	40	28	23	44	2
U18	G19	92	45	90	83	94

Table S5. Percentage of triple stacking interactions detected in the simulations of riboswitches in the unbound state. The first 50 ns of the simulations were excluded from the analysis. The nucleotide sequence is shown in Figure 1.

			Percentage of simulation time [%]				
Base 1	Base 2	Base 3	N1	U14C	U14C$^+$	U15A	A17G
C6	U7	A17	0	0	0	0	25
C6	U8	A17	3	2	9	12	0

Table S6. Hydrogen bonds for analyzed RNA systems in the unbound state. The first 50 ns of the simulations were excluded from the analysis. Only the hydrogen bonds that appear in more than 25% of the analyzed trajectory in at least one system are shown. C12:N3–G19:N1 and C12:O2–G19:N2 hydrogen bonds are stable ($\geq$ 90%). ' defines ribose atoms. * indicates the hydrogen bonds in which an atom is changed as a result of mutation: proton in A17:C2 changes to NH_2, N6 in A17 is replaced by O6. The nucleotide sequence is shown in Figure 1.

		Percentage of simulation time [%]				
Acceptor	Donor	N1	U14C	U14C$^+$	U15A	A17G
G1:O6	C27:N4	76	53	76	75	79
G2:O6	U26:N3	53	58	45	51	60
C3:N3	G25:N1	77	76	74	78	88
C3:O2	G25:N2	82	82	80	82	87
U4:O4	A24:N6	63	68	71	55	62
G5:O5'	U4:O2'	20	25	23	26	12
G5:O6	C23:N4	87	84	87	89	89
G5:OP2	C6:N4	22	23	28	37	9
G5:OP2	C6:O2'	33	45	18	18	2
U7:OP1	U8:O2'	0	0	0	29	0
U8:O2'	C11:N4	0	0	0	1	52
U8:OP1	U7:O2'	11	22	19	28	9
G9:O6	C22:N4	89	88	85	87	88
G9:OP1	A17:N6*	0	8	2	34	0
U10:O2	U21:N3	22	37	19	37	45
U10:O4	U21:N3	55	31	53	20	17
U10:OP2	U8:O2'	0	4	0	4	50
C11:N3	G20:N1	86	84	89	83	89
C11:O2	G20:N2	86	86	89	83	81
U13:O2	U18:N3	26	11	42	43	18
U13:O4	U8:N3	0	0	0	0	37
U14:O5'	U13:O2'	14	26	9	5	10
A16:O3'	A17:C2*	0	0	0	0	37
A16:N7	U14:O2'	5	0	33	12	9
A17:O5'	A16:O2'	25	11	12	21	26
A17:OP2	U14:N3	12	0	63	39	38
U18:O4	U13:N3	58	20	65	57	61
G19:O6	C12:N4	87	82	89	84	86
G20:O6	C11:N4	80	78	83	74	60
U21:O2	U10:N3	52	28	48	18	16
U21:O4	U10:N3	31	51	28	47	68
C22:N3	G9:N1	91	87	87	91	93
C22:O2	G9:N2	89	85	85	88	90
C23:N3	G5:N1	89	90	93	93	95
C23:O2	G5:N2	91	88	92	91	93
A24:N1	U4:N3	71	74	78	60	67
G25:O6	C3:N4	71	72	67	72	80
U26:O2	G2:N1	69	68	65	68	75
C27:N3	G1:N1	82	57	82	82	88
C27:O2	G1:N2	81	56	80	81	86

Table S7. Percentage of Van der Waals interactions between two nucleobases in the simulations of riboswitches in complex with neomycin. The first 50 ns of the simulations were excluded from the analysis. Only the stacking interactions that appear in more than 25% of the analyzed trajectory in at least one system are shown. The following stacking interactions are stable (observed in at least 90% frames of analyzed part of trajectory): G1:G2, G2:C3, G2:C27, C3:U4, C3:U26, U4:G5, U4:G25, G5:G9, G5:A24, G9:U10, G9:C23, U10:C11, U10:C22, C11:C12, C11:U21, C12:U13, C12:G20, U13:G19, A16:U18, U18:G19, G19:G20, G20:U21, U21:C22, C22:C23, C23:A24, A24:G25, G25:U26, U26:C27. The nucleotide sequence is shown in Figure 1.

		Percentage of simulation time [%]				
Base 1	Base 2	N1_NEO	U14C_NEO	U14C$^+$_NEO	U15A_NEO	A17G_NEO
G5	G25	35	37	34	41	35
C6	U7	0	35	18	10	25
C6	U8	27	4	4	10	7
C6	A17	87	93	93	93	82
U7	A17	0	9	5	1	25
U13	U14	95	83	100	56	87
U13	A16	2	37	0	41	13
U14	A16	47	46	59	39	42
U14	U18	89	60	100	55	83
U15	A16	95	80	99	90	89

Table S8. Hydrogen bonds for analyzed RNA systems in the bound state. The first 50 ns of the simulations were excluded from the analysis. Only the hydrogen bonds that appear in more than 25% of the analyzed trajectory in at least one system are shown. C3:N3–G25:N1, C3:O2–G25:N2, C11:N3–G20:N1, C12:N3–G19:N1, C12:O2–G19:N2, G19:O6–C12:N4, C22:O2–G9:N2, C22:N3–G9:N1, C23:N3–G5:N1, C23:O2–G5:N2, A24:N1–U4:N3 hydrogen bonds are stable ($\geq$ 90%). ' defines ribose atoms. * indicates the hydrogen bonds in which an atom is changed as a result of mutation: proton in A17:C2 changes to NH_2, N6 in A17 is replaced by O6. The nucleotide sequence is shown in Figure 1.

		Percentage of simulation time [%]				
Acceptor	Donor	N1_NEO	U14C_NEO	$U14C^+$_NEO	U15A_NEO	A17G_NEO
G1:O6	C27:N4	75	73	76	72	77
G2:O6	U26:N3	73	74	74	75	74
U4:O4	A24:N6	86	82	84	80	77
G5:O2'	A17:N6*	70	75	85	57	1
G5:O6	C23:N4	82	82	83	80	81
G5:OP1	C6:N4	22	26	18	17	3
G5:OP1	A17:C2*	0	0	0	0	31
G5:OP2	C6:N4	32	34	38	37	8
C6:N3	A17:O2'	20	31	16	25	28
C6:O2	A17:O2'	27	21	30	21	11
U7:OP1	C6:O2'	32	19	30	21	4
U8:OP1	U7:O2'	28	19	20	21	18
G9:O6	C22:N4	83	84	83	83	86
G9:OP1	U8:O2'	23	26	12	19	13
U10:O2	U21:N3	56	57	52	57	52
C11:O2	G20:N2	89	89	90	89	90
U13:O2	U18:N3	63	57	65	70	84
A16:N7	U14:O2'	23	13	30	11	38
A17:O5'	A16:O2'	29	5	9	20	12
A17:OP2	U14:N3	70	0	67	46	62
U18:O4	U13:N3	77	77	78	84	84
U18:OP1	A16:O2'	18	26	50	8	36
G20:O6	C11:N4	77	76	78	76	79
U21:O4	U10:N3	79	80	82	80	82
G25:O6	C3:N4	86	87	87	87	87
U26:O2	G2:N1	81	81	81	82	82
C27:N3	G1:N1	83	82	84	80	86
C27:O2	G1:N2	81	81	83	79	85

Table S9. Neomycin interactions with RNA. The first 50 ns of the simulations were excluded from the analysis. The percentage of occurrence of hydrogen bonds with ammonium groups in neomycin is calculated as a sum of hydrogen bonds with different protons. Only the hydrogen bonds that appear in more than 10% of the analyzed trajectory in at least one system are shown. * indicates the hydrogen bonds in which an atom is changed as a result of mutation: N6 in A17 is replaced by O6. The nucleotide sequence and structure of neomycin are shown in Figure 1 and Figure S1, respectively.

		Percentage of simulation time [%]				
Acceptor	Donor	N1_NEO	U14C_NEO	U14C^{+}_NEO	U15A_NEO	A17G_NEO
C3:OP1	NEO:O12	13	10	10	5	3
C3:OP1	NEO:O13	22	16	19	5	1
U4:OP1	NEO:O12	2	1	18	1	3
U4:OP2	NEO:O10	7	8	5	31	15
U4:OP2	NEO:O12	14	10	15	3	1
U4:OP2	NEO:N5	32	41	20	35	40
G5:OP2	NEO:O1	1	0	0	18	0
G5:OP2	NEO:O10	50	64	41	41	57
G9:OP1	NEO:N3	38	42	56	57	37
U10:O4	NEO:N3	65	65	67	52	11
A17:N6*	NEO:N3	3	3	3	2	55
U18:OP2	NEO:N6	9	6	6	12	31
G19:O6	NEO:O8	65	67	67	62	64
G19:OP1	NEO:O12	2	3	3	17	0
G19:OP1	NEO:O13	9	11	5	20	30
G19:OP2	NEO:O1	89	86	79	64	85
G19:OP2	NEO:N6	18	12	18	12	36
G19:N7	NEO:N4	42	39	40	32	42
G20:O6	NEO:N2	46	47	50	43	48
G20:N7	NEO:N4	61	57	54	46	49
U21:O4	NEO:N2	42	44	41	46	43
